# Immunoreactive Microenvironment Modulator GBP5 Suppresses Ovarian Cancer Progression by Inducing Canonical Pyroptosis

**DOI:** 10.7150/jca.94616

**Published:** 2024-05-05

**Authors:** Chang Zou, Jiacheng Shen, Fangfang Xu, Yingjun Ye, Yuanyuan Wu, Shaohua Xu

**Affiliations:** Shanghai Key Laboratory of Maternal Fetal Medicine, Shanghai Institute of Maternal-Fetal Medicine and Gynecologic Oncology, Shanghai First Maternity and Infant Hospital, School of Medicine, Tongji University, Shanghai 200092, China.

**Keywords:** GBP5, pyroptosis, JAK/STAT pathway, ovarian cancer, macrophage

## Abstract

Ovarian cancer has the highest mortality among gynecological malignancies, and exploring effective strategies to reverse the immunosuppressive tumor microenvironment in patients remains a pressing scientific challenge. In this study, we identified a pyroptosis-related protective factor, GBP5, which significantly inhibits the growth of ovarian cancer cells and patient-derived ovarian cancer organoids, impeding the invasion and migration of ovarian cancer cells. Results of immunohistochemistry and external single-cell data verification were consistent. Further research confirmed that GBP5 in ovarian cancer cell can induce canonical pyroptosis through JAK2/STAT1 pathway, thereby restraining the progression of ovarian cancer. Interestingly, in this study, we also discovered that ovarian cancer cells with high GBP5 expression exhibit increased expressions of CXCL9/10/11 in a co-culture assay. Subsequent immune cell infiltration analyses revealed the remodeling of immunosuppressive microenvironment in ovarian cancer patients, characterized by increased infiltration and polarization of M1 macrophages. External immunotherapy database analysis showed profound potential for the application of GBP5 in immunotherapy strategies for ovarian cancer. Overall, our study demonstrates that the protective factor GBP5 significantly inhibits ovarian cancer progression, triggering canonical pyroptosis through the JAK2-STAT1 pathway. Driven by its pro-inflammatory nature, it can also enhance M1 macrophages polarization and reverse immunosuppressive microenvironment, thus providing new insights for ovarian cancer treatment.

## Introduction

Ovarian cancer (OC) has the highest mortality rate among gynecological malignancies, with epithelial ovarian cancer (EOC) contributing the most, accounting for 90% of all ovarian malignancies cases 1. Immunotherapy has been highly expected in the past decade. Although immunotherapy functions by activating intra-tumoral immune cells with few side effects and high efficiency, it fails in ovarian carcinoma. The clinical responsivity of OC patients receiving immune checkpoint inhibitors (ICIs) monotherapy remains 10-15%. Our previous research revealed that nearly half of OC patients exhibited immune-suppressed tumor microenvironment (TME) accompanied by immune response blockage, thus leading to poor effectiveness of immunotherapy. In immunosuppresive TME, tumorous antigen presentation was blocked due to the recruitment and activation of immunosuppressive cells/molecules, this phenomenon is marked as a “cold tumor”^2^. To “heat” TME is crucial for improving ICIs effectiveness 3-5.

Pyroptosis is a type of programmed cell death (PCD), distinguished from apoptosis. It's divided into inflammasome-dependent and -independent types. The former comprises canonical and non-canonical ways 6. The self-cleavage and activation of Caspase-1 (CASP1) serves as a canonical pyroptosis hallmarker, leading to the exposure of cell-toxic N-terminal in gasdermin D (GSDMD-N) protein 7, 8. Afterwards, cell rupture occurs accompanied by the release of proinflammatory cytokines (IL-1β, IL-18) and immunogenic cell components. Previous studies have shown that cancer cell pyroptosis activation leads to higher level of intratumor immune cell infiltration, antitumor immunity activation, and tumor growth suppression 9. IL-1β released during cancer cell pyroptosis serves crucially in inducing dendritic cell (DC) maturation, the recruitment and activation of natural killer (NK) cells and CD8^+^ T cells, and the inhibition of immunosuppressive regulatory T cells (Tregs) differentiation, all of which are involved in reversing immunosuppressive microenvironment 10, 11. Therefore, pyroptotic cancer cell death, an immunogenic form of cell death to stimulate antitumor immunity and reprogram the tumor immune microenvironment (TIME) out of the immunosuppressive state, serves as a mechanism for potential and effective strategies to enhance ICIs sensitization. Although several studies have confirmed the role of pyroptosis in cancer therapy, there is limited research on the association between pyroptosis and antitumor immunity in OC patients. Further investigation into the role of pyroptosis in enhancing antitumor immunity of OC patients exhibits promise for clinical purposes.

We aimed to discover effects exerted by pyroptotic OC cells on neighboring immune cells within the TIME. Among immune cell types exhibiting observable change in infiltrating level, M1-type macrophage gained our attention due to its pro-inflammatory and antitumor characterisms supported by previous researches, with its role as the only protective factor among immune cell types to OC according to bioinformatic analysis conducted by us. İn the early stage of TIME formation, M1-like macrophages work as tumor growth suppressor 12, 13, partly by recruiting CD8^+^ T cell and NK cells through antigen presentation to the T-cell receptor (TCR) 14, also driving the release of chemokines (C-X-C motif chemokine ligand (CXCL) 9/10/11) derived from tumor cells to recruit NK cells 15. The characteristic of pyroptosis about promoting antigenic substance release (from tumor cells) may induce an inflammatory response driven by M1 macrophages. On the other hand, M1 macrophages are capable of secreting pyroptosis inducer interferon-gamma (IFNγ) 16-18. The above evidence is sufficient to support the close relation between pyroptotic products and M1 macrophage activation.

In this study, we intersected the lists of biomarkers associated with M1 macrophage polarization and pyroptosis regulators, and subsequently conducted LASSO penalized COX analysis. Among indicators that effectively predicted OC patients survival, guanylate-binding protein 5 (GBP5) caught our attention for its strongest correlation with M1-type macrophage infiltrating score. GBP5 appeared to play a benign role in OC, it is expressed significantly higher in non-malignant cells and early-stage OC tissues. *In vitro* experiments indicated that GBP5 inhibited the proliferation, migration, and invasion of OC cells. Based on patient-derived ovarian cancer organoid (PDOCO) model, we observed that GBP5 potentially impeded OC growth at organ level. Moreover, the modulative effect of GBP5 on OC cell pyroptosis was mechanistically disclosed. There was a clue that gene set enrichment analysis (GSEA) revealed the positive connection between the high GBP5 expression and the activation of the JAK-STAT pathway. Through *in vitro* assays, we confirmed that high expression of GBP5 induced OC cell canonical pyroptosis through JAK2-STAT1-CASP1 axis. Crucially, according to bioinformatic analyses, GBP5 reprogrammed TIME in an immunoreactive way, especially relying on M1 macrophage activation. The pan-cancer analysis presented a common positive correlation between M1-type macrophage polarization and GBP5 expression. Mø kept in an OC cell-macrophage coculture system showed an enhancing polarization trend towards classical-type macrophage with upregulated secretion of inflammatory factors, and the increasing transcriptional levels of chemokines CXCL 9/10/11 in GBP5-overexpressed OC cells gave a possible explanation. Being analyzed from the immunophenoscore (IPS) distribution and external public immunotherapy database, an observed increase in the expression of programmed cell death 1 ligand 1 (PD-L1) driven by high expression of GBP5 endowed its potential role as a survival indicator for tumor patients receiving anti-PD1 and anti-PD-L1 therapy. In summary, the extensive potential application value of GBP5 has been suggested.

## Materials and Methods

### Bioinformatic Analyses

#### RNA-seq Data and Clinical Information Resources

Statistical data were processed using the R tool (Version 4.2.2). Transcriptional profiles and clinical information for 451 OC samples utilized in this research were sourced from The Cancer Genome Atlas (TCGA) database, UCSC Xena website, and dataset GSE63885 in Gene Expression Omnibus (GEO) database. Additionally, single-cell RNA sequence (scRNA-seq) data for 8 OC samples were obtained from GSE217517, ensuring the percentage of mitochondrial gene remained below 10% for each sample 19. To address batch effects, the R package “sva” was applied 20. Consequently, a total of 440 samples met the inclusive criteria. Samples with complete clinical information, overall survival (OS) period exceeding one month, pathological diagnosis of ovarian serous cystadenocarcinoma, living status, and recorded cause of death attributed to OC when clinical outcomes were noted as “death” were included. The full names of 38 types of cancers were listed in **Table S1**.

### Gene Ontology (GO), Kyoto Encyclopedia of Genes and Genomes (KEGG), GSEA Analyses

GO and KEGG analyses were conducted using the R packages “clusterProfiler”^21^ and “enrichplot”. Entries with an adjusted q-value < 0.05 and p-Value < 0.05 were considered significant. Databases used in GSEA analysis included “Hallmarks v7.5 symbols”, “c2.cp.kegg v7.5 symbols”, supported by GSEA 4.1.0 software 22. A NOM p-Value and FDR q-Value less than 5% were considered significant.

### Statistics Analyses

Statistical analyses of bioinformatics data were completed by R software (version 4.2.2), while experimental data underwent scrutiny using SPSS 25.0 statistical software. Significance was established at a p value < 0.05. In the graphical representations, significance levels were denoted as follows: *p < 0.05, **p < 0.01, ***p < 0.001, and ****p < 0.0001. Univariate COX combined with LASSO penalized COX analyses were conducted using the “glmnet” 23 and “survival” 24 packages in R. Consensus clustering was performed, with the optimal K determined by proportion of ambiguous clustering (PAC) set at 3, using the ConsensusClusterPlus package. Survival analyses and graphical visualization of the outcomes were conducted using the R packages “survminer” and “survival” 25. Methods for evaluating the immune cell infiltration in this study included CIBERSORT, XCELL, EPIC, single sample GSEA (ssGSEA), TIMER, and quanTIseq, all implemented using the R package IOBR 26. The ESTIMATE algorithm was employed for immune and stromal scores using the R package ESTIMATE 27. The analysis of sc-RNA seq relied on the R packages “Seurate” 28, “UCell” 29 and “SingleR”. All figures resulting from bioinformatic analyses in this article were designed using the R tool with packages such as “ggplot”, “ggplot2”, “pheatmap”, “ggpubr”, “ggExtra”, and “ggradar”. Statistical charts were generated using GraphPad Prism 8 (GraphPad, La Jolla, CA, USA). The processing and quantization of cell experiment results were carried out using ImageJ software (Version 1.53).

### Website Tools

This research benefitted from various website tools, including Sangerbox3.0 website tool 30, multi-omics database LinkedOmics 31, Tumor Immunotherapy Gene Expression Resource database 32, 33, Tumor-Immune System Interactions DataBase 34 (TISIDB). Information on immunophenoscore (IPS) was obtained from The Cancer Immunome Atlas database 35, 36 (TCIA). The list of pyroptosis markers and specific gene information on GBP5 was sourced from GeneCards website.

### Experiment

#### The Culture, Transfection of Cells and Organoids and other Treatments

The human leukemic cell line (Thp1), Jurkat cell line, OC cell lines (SKOV3, HEY, OVCAR3) were procured from the American Type Culture Collection, Manassas, VA, United States. SKOV3, OVCAR3, Jurkat and THP-1 cells were cultured in RPMI 1640 medium, while HEY cells were cultured in DMEM with high glucose (Jiangsu KeyGEN Bio TECH Corp., Ltd., China), supplemented with 10% fetal bovine serum (FBS, Biological Industries) and 1% penicillin/streptomycin (P/S, Wuhan Servicebio Technology, China) at 37 ºC, 5% CO2.

The transfections in SKOV3, HEY and Jurkat cells were conducted with 700 ng/ml pcDNA3.1(+)-Flag-GBP5-overexpression plasmid (Public Protein/Plasmid Library) for 12 hours using Lipofectamine 2000 (Lipo2000) reagent (Invitrogen, USA). The same dose of Lipo2000 was applied to the normal control cells. Fludarabine (MCE, HY-B0069) was chosen as STAT1 inhibitor used in rescue experiments with a concentration of 5 μM, OC cell cohorts without STAT1 inhibition were additionally treated with dimethyl sulfoxide (DMSO) for 24h at the same dose as fludarabine, before transfection. The supernatant of culture medium were collected. Transfection on organoids followed the protocol stated in the research by Dekkers JF *et al.* 37. OC organoids used in all the experiment of this study have undergone up to and including 6 passages.

An human OC cell-immune cell (Macrophage or T cell) co-culture system was established in this research. Thp1 cells (5×10^4^ cells/100ml) were induced into Mø by treatment with 100 ng/ml PMA (phorbol 12-myristate-13-acetate, MCE) for 48 hours. The adhesion of M0 macrophages to the well bottom was observed, followed by a change in the culture medium with 100 ng/ml LPS (lipopolysaccharide, Peprotech) for 48 hours to obtain M1 macrophages. Jurkat cells were placed at the well bottom at nearly 2.5 × 10^6^ cells/ml. Cell line HEY got planted on the transwell device (Corning, USA) with a 0.4 mm porous membrane, placed upon the six-well plate in a concurrent liquid environment with Mø and Jurkat, respectively, and kept for 24h. After the coculture, M0/M1 macrophages and Jurkat T cells were collected for RNA extraction.

#### RNA Extraction and qRT-PCR

RNA extraction from cell lines was performed using RNAiso Plus reagent (Takara Bio, Japan) following the product recommendations. Subsequently, the extracted RNA was reverse-transcribed into complementary DNA (cDNA) using the 5X ALL-IN-One RT Master Mix kit (Applied Biological Materials Inc). Purity and concentration assessment were carried out using the NanoDrop 2000 (Thermo Scientific, USA). qRT-PCR was conducted with TB Green Premix Ex Taq™ kit (Takara Bio). Primer sequences for GBP5, JAK (janus kinase) 2, signal transducer and activator of transcription (STAT)1, STAT2, STAT3, CASP1, CASP3, GSDMD, GSDME, IL-10, transforming growth factor-β (TGFB)1, IL-1β, CD80, TNF-α, C-C Motif Chemokine Ligand (CCL)5, C-C Motif Chemokine Receptor (CCR)5, CCR7, C-X-C Motif Chemokine Ligand (CXCL)9, CXCL10, CXCL11, Granzyme B (GZMB), CD69, and Glyceraldehyde-3-Phosphate Dehydrogenase (GAPDH) are provided in **Table S2**. The obtained results were normalized to GAPDH expression and processed using relative threshold cycle method in 2^-ΔΔCt^.

#### CCK8 Assay

The CCK-8 test was conducted using the Cell Counting Kit-8 kit (Dojindo Laboratories, Japan) to assess cell viability following specific treatments. SKOV3 and HEY cells were respectively plated in a 96-well plate at the density of 2000 cells/well. Every 24 hours, viable cells were measured by adding the CCK8 reagent and culturing for 2 hours at 37 °C in an incubator, subsequently detecting the optical density (OD) value. The experiment was performed in triplicate for each condition.

#### Transwell Invasion Assay

SKOV3 and HEY cell lines were planted on transwell devices, immersed in 150μl culture medium, and placed upon a 24-well plate. Lower chamber was filled with 800μl DMEM with high glucose supplemented with 20% FBS. After a 24-hour incubation at 37 °C with 5% CO2, cells on the upper surface of the transwell chamber bottom were removed using a cotton swab. The membranes were fixed with methanol for 15 minutes, followed by crystal violet staining for 30 minutes and a subsequent washing in phosphate buffered saline (PBS). Five visual fields were randomly selected under microscope, and the representative result was presented. Cell counting for successful invasion was performed using ImageJ. Differential analysis of cell numbers was conducted using a Student t-test, with statistical signicance set as P < 0.05.

#### Wound Healing Assay

SKOV3 and HEY cell lines were plated in 12-well plates and incubated with standard culture medium for 24 hours until reaching confluence. The cell layer was then carefully scratched using a 200μl pipette tip, followed by two PBS washes to remove floating cells and debris. The culture medium was replaced with serum-free medium. Marks were made on the plate's cover, and images of the scratch healing were captured at 0, 24, and 48 hours at specific positions under a inverted microscope, illustrating representative results. The migration capacity of OC cells was quantified based on the percentage of changed area, calculated as (0h Scratch Area - Observed Scratch Area) / 0h Scratch Area × 100%. The scratch area calculation relied on ImageJ, derived from observations at a minimum of three different spots. Differential analysis of the changed area proportion was performed using the Student t-test, with statistical signicance set as P < 0.05.

#### 5-Ethynyl-2'-deoxyuridine (EdU) assay

The Click-iT EdU-555 Kit (Servicebio, China) was utilized according to the official protocol for EdU staining. SKOV3 and HEY cells were seeded in 96-well plates at a density of 4000 cells/well for 24 hours, followed by EdU treatment and incubation at 37 °C with 5% CO2 for 2 hours. OC cells were fixed with 4% paraformaldehyde, and permeabilization was carried out with 0.3% TritonX-100. The click solution was configured based on the manufacturer's recipe, and cells were treated with the solution in a light-protected environment for half an hour. Cell nuclei were counterstained with 4′,6-diamidino-2-phenylindole (DAPI) (Servicebio, China). Newly-proliferated cells during the 2-hour culture period were observed and captured using an inverted fluorescence microscope (Carl Zeiss, Germany). The experiment was performed in triplicate, and representative results were presented in images.

#### TUNEL assay

Cell climbing sheets loaded with HEY cells were prepared in a 6-well plate, followed by TUNEL staining using the Fluorescein (FITC) Tunel Cell Apoptosis Detection Kit (Servicebio, China) according to supplier's protocol. The cell nuclei were fluorescently stained with DAPI (Servicebio, China). Pyroptosis cells were visualized under an inverted fluorescence microscope (Carl Zeiss, Germany), and representative results were selectively presented.

#### Immunohistochemistry (IHC)

Twelve pathological samples of ovarian serous cystadenocarcinoma were collected from Shanghai First Maternal and Infant Hospital for inclusion in the research cohort. The diagnoses were based on the latest International Federation of Gynecology and Obstetrics (FIGO) guidelines, with 6 samples in stages I-II and 6 smples in stages III-IV. Informed consent was obtained for the acquisition of clinical samples, and the study was approved by Medical Ethics Committee of Shanghai First Maternal and Infant Hospital (the ethical certification number: KS2142).

The primary antibody against GBP5 (1:100, PHC6203, abmart, China) was applied overnight at 4 °C, followed by incubation with a secondary antibody (PK-8501, Vector Lab, USA) for 1 hour. The Rabbit IgG mini-PLUS Kit was utilized to visualize the DAB complex (PK-8501, Vector Lab, USA). Hematoxylin was used for nuclear staining. The final sections were examined, and representative results were selectively presented in images.

#### Generation, Culture, and Multiplex Immunofluorescence (MIF) of OC Organoids

Fresh ovarian cancer tissues were cut into 3-5mm^3^ pieces for organoid derivation. The tissue was immersed by 10ml AdDF+++ (Advanced DMEM/F12 containing 1x Glutamax, 10mM HEPES, and antibiotics) and digested in 10ml AdDF+++ with 1mg/ml collagenase IV (Sigma-Aldrich) by shaking at a frequency of 150 r.p.m. for 20 minutes. After adding 10% FBS (Biological Industries) for neutralization, the supernatant was filtered through a 70 μm cell stainer (Biosharp). The successully filtered fractions were pooled and centrifuged at 250g for 5 minutes at 4 °C. The erythrocyte-containing pellet was immersed in 2 ml red blood cell lysis buffer (Shanghai Fusheng Biotech) to lyse for 5 minutes at room temperature, followed by a wash in 10 ml AdDF+++ and centrifugation at 250g for 5 min until the erythrocytes were entirely cleared. The pellet was then cleaned once with AdDF+++.

The cell pellet was suspended in cold Cultrex growth factor-reduced BME type 2 (3533-001-02, R&D), and the organoid clumps were seeded in 40 µl BME droplets, placed on a pre-warmed 24-well plate, and left for 30 minutes at 37 °C until solidified. Adding 0.5 ml of appropriate organoid medium (showed in **Table S3**) to BME stabilization, the model was kept in a 37 °C/5% CO2 incubator. Organoid counting and size measurement was accomplished using ImageJ.

The MIF staining protocol for OC organoids is as follows: I. Wash and collect OC organoids with pre-cold PBS; II. Transfer the organoids to 4% paraformaldehyde and fix at room temperature for 30 minutes; III. Wash the organoids three times in 3% BSA-PBS for 5 min each time; IV. Transfer the organoids to 0.2% Triton X-100 solution and incubate at room temperature for 20 min; V. After repeating step III, transfer the organoids to 1% BSA solution for blocking and incubate at room temperature for 4 hours; VI. After repeating step III, transfer the organoids to a mixture of primary antibodies (diluted in 1% BSA) and incubate overnight at 4 °C. The primary antibodies include PAX8 Polyclonal antibody (1:300, proteintech, 10336-1-AP), E-Cadherin (24E10) Rabbit mAb (1:300, CST, 3195), pan-Cytokeratin (1:300, Santa Cruz, sc-8018), Ki67 antibody (1:300, Santa Cruz, sc-23900); VII. Wash the organoids three times in 0.05% Tween 20-PBS for 5 min each time; VIII. Transfer the organoids to fluorescent secondary antibodies (anti-Rabbit 647, 1:500, CTX, CST; anti-Mouse 488, 1:500, CTX, CST) and incubate at room temperature for 2 hours in the dark; IX. After repeating step VII, transfer the organoids to DAPI (Servibio, G1012) solution to stain cell nuclei; X. After repeating step VIII, Resuspend the organoids in anti-fading mounting medium, place them on a confocal dish, and capture images using confocal microscopy.

#### Western Blotting

A mixture containing RIPA buffer, protease, and phosphatase inhibitors (TargetMol, America) was prepared for cell lysing. The protein supernatant was extracted after ultrasonic homogenization and centrifugation to remove impurities. The concentration of the extracted protein was measured using BCA (Beyotime, China). Samples were heated at 100 °C for 10 minutes, loaded onto 20% sodium dodecyl sulfate-polyacrylamide gel electrophoresis (SDS-PAGE, Servicebio, China), and transferred to PVDF membranes. Membranes prepared for phosphorylated protein detection were blocked with TBST containing 3% BSA, and the rest membranes were blocked with 5% non-fat milk for 2 hours. Blocked membranes were incubated with the primary antibody at 4 °C overnight, followed by incubation with anti-Rabbit or anti-Mouse secondary antibodies for 2 hours at room temperature. In the final step, enhanced chemiluminescence (ECL) reagent (EpiZyme, China) was applied to visualize the protein bands. The primary antibodies used in this research were as follows: GBP5 (1:500, PHC6203, Abmart), Phospho-STAT1 (p-STAT1, Tyr701) Antibody (1: 1000, AP0054, ABclonal), Total and Cleaved Caspase 1 Antibody, Total and cleaved N-terminal GSDMD (GSDMD-N) Antibody (1:500, P30823, Abmart), PD-L1 Antibody (1:1000, M033179, Abmart), β-Actin Rabbit mAb (High Dilution) (1:10000, AC026, Abclonal). Secondary antibodies were purchased from ABclonal, China.

#### Enzyme-Linked Immuno-Sorbent Assay (ELISA)

In the rescue experiment, the IL1β Elisa kit (AB0908, Abclonal) was utilized to assess pyroptotic cell death. The procedure adhered to the supplier's protocol, where we constructed the standard curve using a four-parameter logistic (4-PL) model and conducted the concentration detection.

## Results

### Identification of Prognostic Biomarkers for M1 Macrophage Infiltration Associated with Pyroptosis in Ovarian Cancer

**Figure 1:**
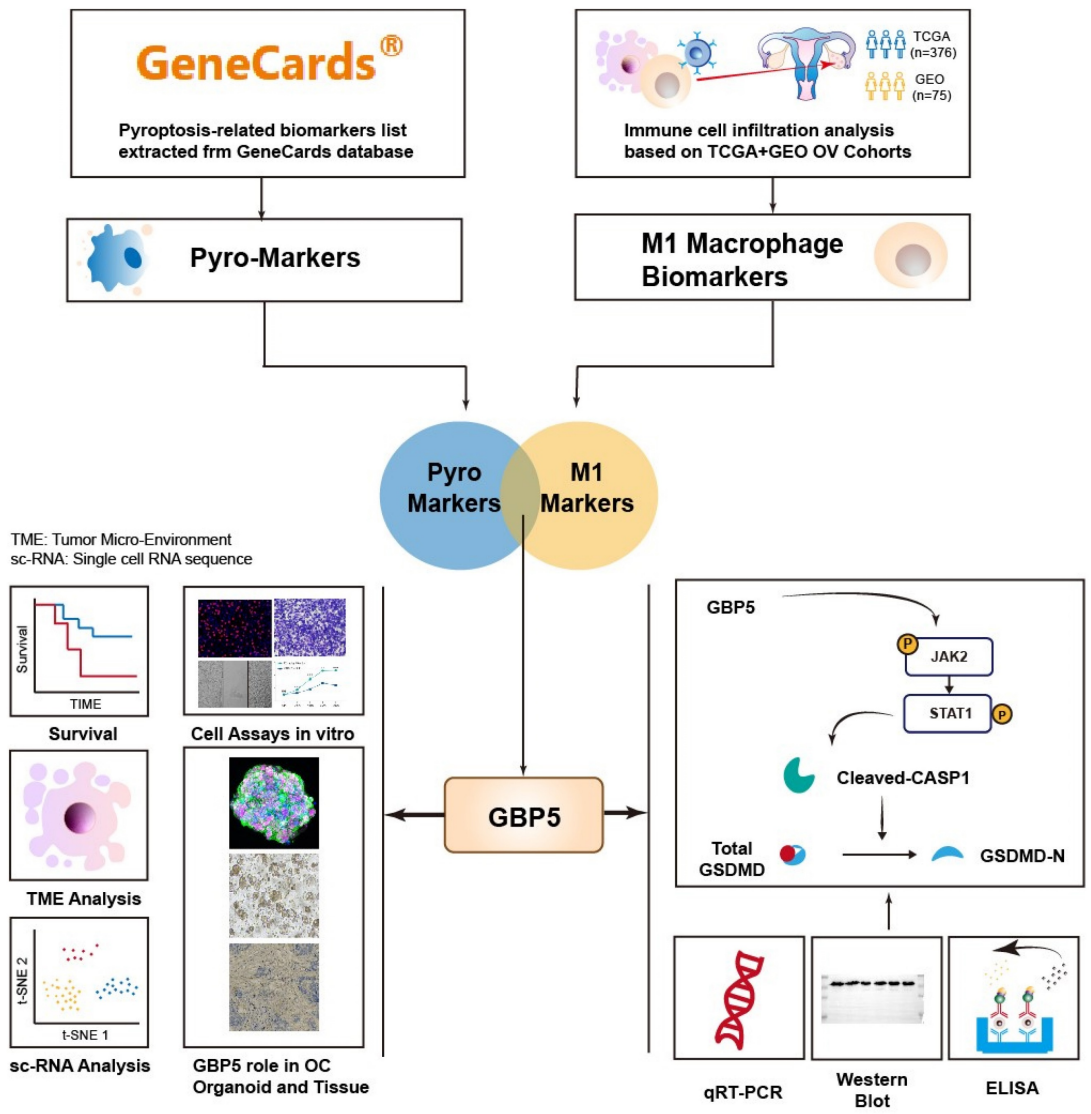
The flowchart illustrating the complete process of this work.

**Figure 2:**
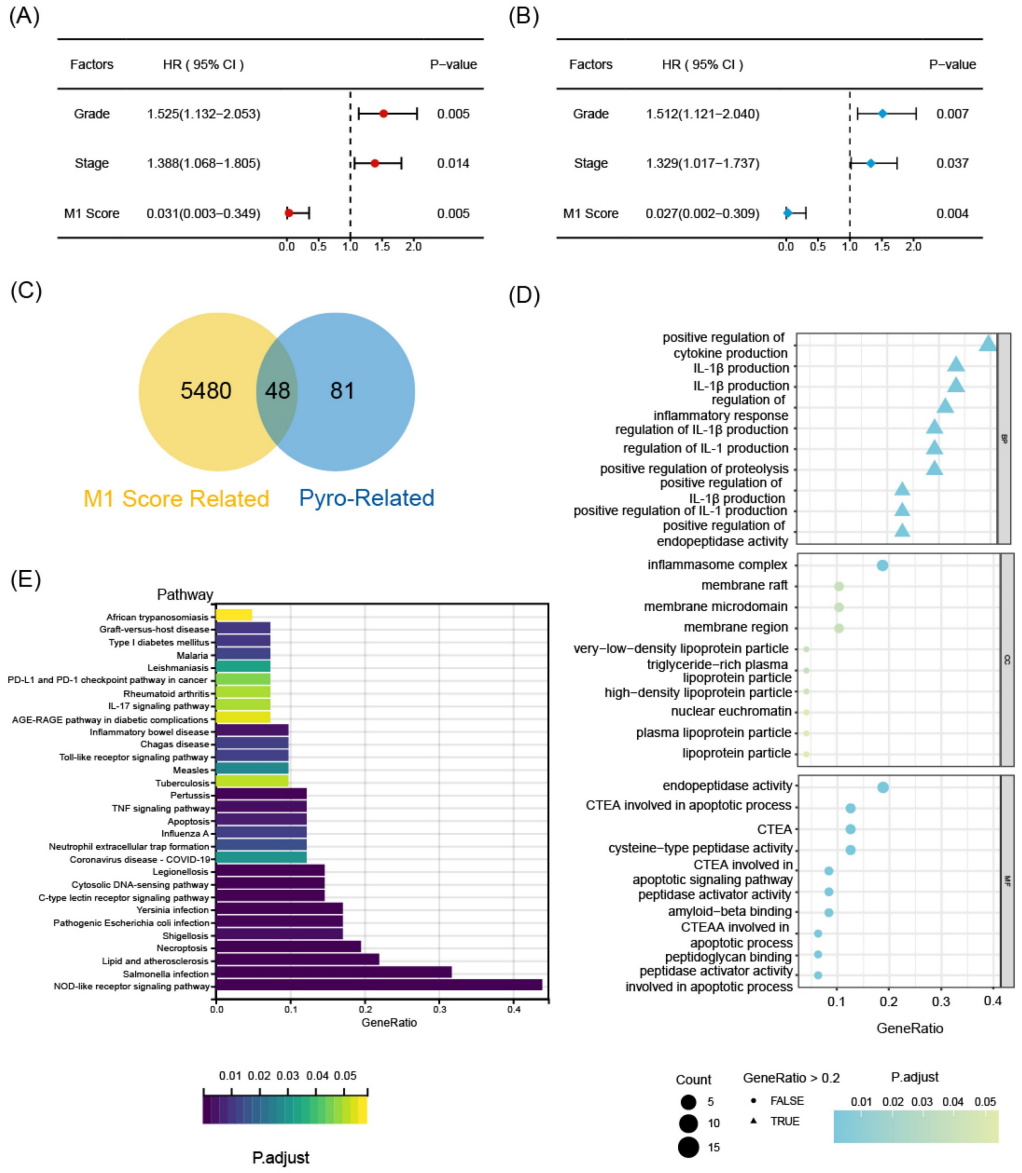
The selecting process of OC-prognostic pyro-M1 biomarkers. The (A) univariate COX analysis and (B) multivariate COX analysis on M1-macrophage score and grade, stage were illustrated. (C) the 5528 DEGs between high- and low- M1 macrophage cohorts were intersected with 129 genes involved in the process of pyroptosis, and the selected 48 candidates were sent for further research. (D) GO and (E) KEGG analyses were completed between the 48 candidates.

The flowchart of this work is shown in **Figure 1**. The CIBERSORT algorithm was employed to quantify the infiltration of twenty-one types of immune cells in 440 OC patients, presenting the results as infiltrating scores. Along with clinical features, grade and FIGO stage, the 21 infiltrating scores in each patient were considered as variables and potential independent prognostic indicators for subsequent analyses. Among them, tumor grade, FIGO stage, and the infiltrating score of M1-type macrophage were identified as significantly evaluable factors, accoding to the results of subsequent uni- and multi-variate Cox regression analyses. This observation aligned with the common understanding, that FIGO stage typically serves as a risk factor for cancer patients, while M1 macrophages are commonly considered protectors against the immune silence induced by malignancy due to their pro-inflammatory nature (**Figure 2A, B**).

Encouraged by discoveries above, our focus shifted to M1 macrophages. OC patients were grouped into high- and low-M1 cohorts, with the cutoff set as the median value of M1-type macrophage infiltrating scores. The differential expression analysis revealed differentially expressed genes (DEGs) between the transcriptional profiles of two cohorts, 48 out of 5528 DEGs simultaneously served as M1-score-related markers and pyroptosis regulators, which were defined as pyro-M1 biomarkers (**Figure 2C**). The enrichment analyses (KEGG and GO) unveiled high enrichment of pathways related to IL-1 production and regulation, especially IL-1β, well-known for its roles as inflammatory molecule and pyroptosis marker. The enrichment in pathways related to cysteine-type endopeptidase (CTE) and CTE activator activities were also observed, with CTE known to participate in the apoptotic process (**Figure 2D**). Furthermore, other enriched pathways, which are related to responses to various microbial infections, pathogen sensing, and the production of inflammatory participants, were predominantly associated with inflammatory diseases or intricately linked to assembling inflammasomes (**Figure 2E**).

**Figure 3:**
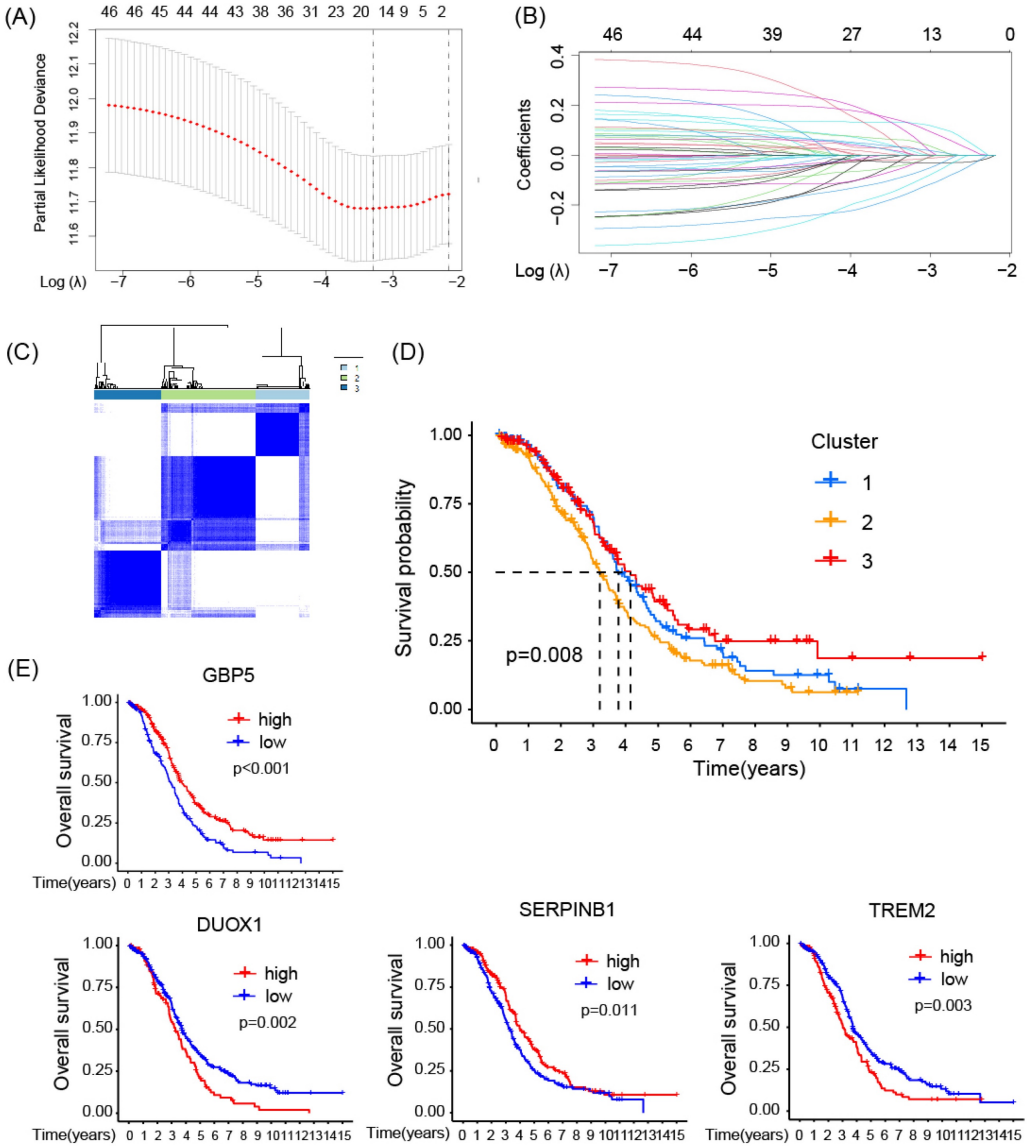
The prognostic function of the selected biomarkers.** (A, B)** The least absolute shrinkage with 10-folded cross-validation, conducting candidate shrinkage. **(C)** Heatmap of the consensus clustering.** (D)** The Kaplan-Meier survival analysis between the 3 consensus clusters. **(E)** The result of single gene K-M survival analysis concentrated on each pyro-M1 biomarker.

To identify statistically significant prognostic biomarkers for OC, we employed univariate Cox regression analysis combined with LASSO-COX regression. This process involved the elimination of genes with similar expression patterns, retaining representative ones with the most effective prognostic capability. Ultimately, four candidate biomarkers were selected (**Figure 3A, B**).

Consensus clustering analysis was performed on the expression profiles of 4 pyro-M1 biomarkers: GBP5, DUOX1, SERPINB1, TREM2. Based on their genetic expression patterns, OC patients were stratified into 3 subgroups (optimal K=3). Boundaries of consensus matrix among subgroups were clear and sharp, as illustrated in the heatmap (**Figure 3C**). Notably, the overall survivals (OS) significantly varied among the subgroups, as expected (**Figure 3D**). Accordingly, Group 3 exhibited the best survival outcome among the three clusters, especially when compared with Group 2 (p = 0.008). We further concentrated on the immune-related genes expression pattern of these patients (**Figure S1A**), it seemed Group 3 showed an up trend of tumor-associated macrophages (TAMs) polarization and a slight increase in enrichment of CD8^+^ T cells, while Group 2 exhibited a higher level of M2 macrophage activation compared with Group 3. The single gene Kaplan-Meier survival analysis revealed that each of the four biomarkers can serve as an independent prognostic indicator for OC. High expressions of GBP5 and SERPINB1, as well as low expressions of DUOX1 and TREM2, were associated with better survival in OC (**Figure 3E**).

### High Expression of GBP5 Indicates Immunoreactive Tumor Microenvironment

**Figure 4:**
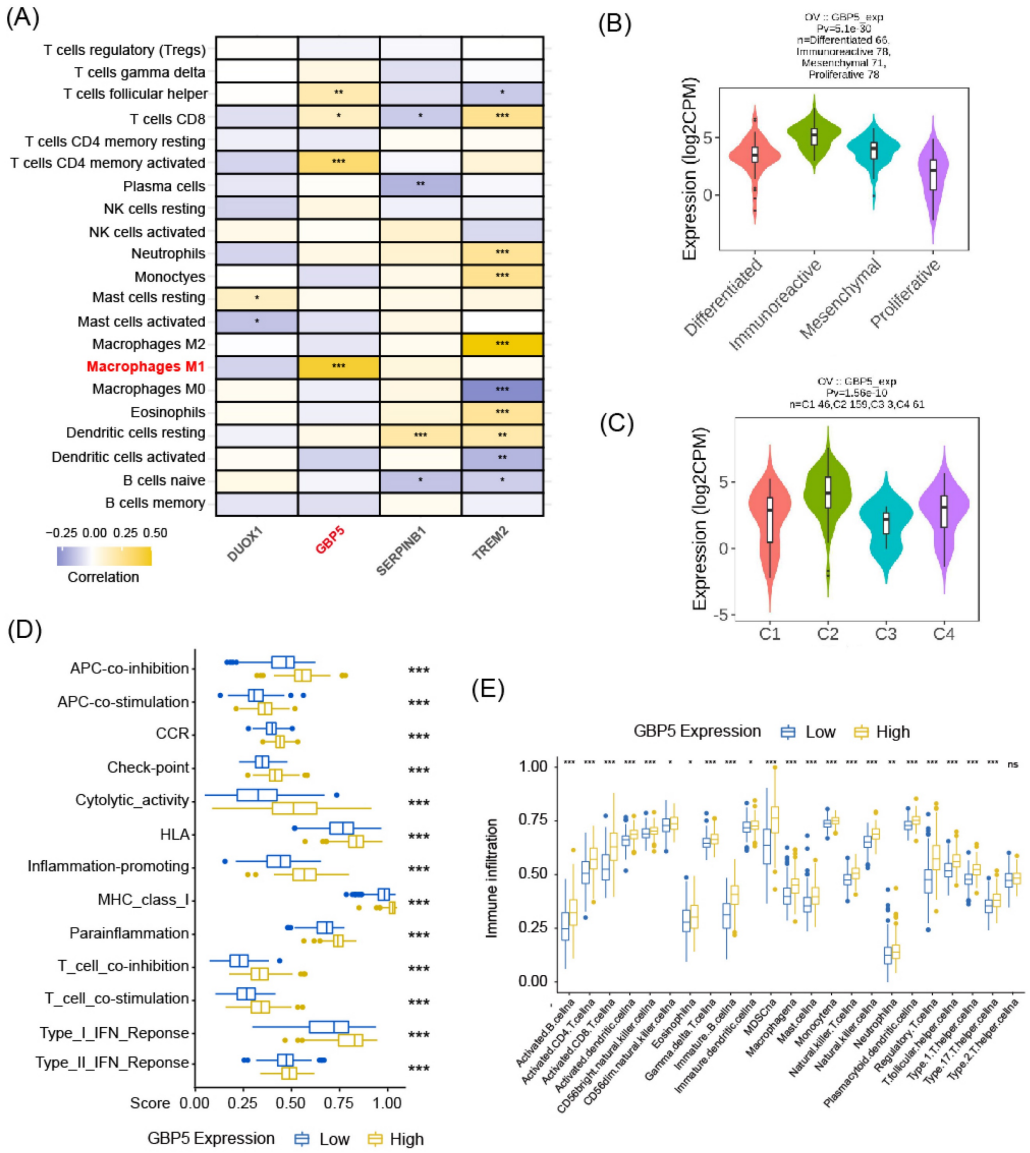
The correlation between GBP5 expression and immune cell infiltration. **(A)** The heatmap shows the Spearman correlation between CIBERSORT scores and expression levels of the prognostic biomarkers. The significance is shown by “*,” “*” stands for p-Value < 0.05, “**” as p-Value < 0.01, “***” as p-Value < 0.001, blocks without “*” mark means result without significance. **(B)** GBP5 expression level in various ovarian cancer molecular subtypes. The sample capacity and the p-Value are listed at the figure top. **(C)** GBP5 expression level in various immune subtypes. OC samples are divided into four subtypes, including C1 (wound healing), C2 (IFN-gamma dominant), C3 (inflammatory), and C4 (lymphocyte depleted). The sample capacity and the p-Value are listed at the figure top.** (D)** The ssGSEA algorithm illustrated the enriched immune function pathways. “***” means p-Value < 0.001.** (E)** The result of ssGSEA analysis, to show the distribution of immune cells divided by the expression of GBP5. “*” means p-Value < 0.05, “**” means p-Value < 0.01, “***” means p-Value < 0.001, “ns” means the result lacks significance.

As aforementioned, M1-type macrophage contributed to the pro-inflammatory microenvironment in various situations, and the enrichment of M1 macrophage indeed predicted a better prognosis in OC patient cohort, as demonstrated in our previous research. Spearman correlation analysis results, as demonstrated in **Figure 4A**, revealed that among all the pyro-M1 markers, GBP5 expression exhibited the highest positive correlation with M1 macrophage infiltrating score. Research by Konecny GE, *et al.* 38 categorized OC into 4 molecular subtypes: differentiated, immunoreactive, mesenchymal, proliferative. Researchers analyzed the distribution of GBP5 expressions in OC patients (corresponding information acquired from TCGA database grouped into these 4 molecular subtypes, it was found that patients with immunoreactive-subtype OC exhibited the highest GBP5 expression levels, and the immunoreactive subtype commonly presents best survival status (**Figure 4B**). Similarly, when classifying OC patients based on immune landscapes 39, the IFN-γ dominant subtype (C2) exhibited the highest GBP5 expression, and this subtype is commonly marked by high levels of macrophages and CD8^+^ T cells activations (**Figure 4C**). Similar results were observed not only in OC but also in other 25 cancer types (cancer types without C2-type samples were excluded in this analysis) (**Figure S1B**), indicating that the positive correlation between high GBP5 expression and the C2 phenotype can be considered a pan-cancer commonality. Immune function pathways were highly activated in the patient group with high GBP5 expression, as assessed by ssGSEA, and were notably associated with inflammation promotion and IFN response possibly due to GBP5's IFN-γ-inducible characteristic (**Figure 4D**). Moreover, being quantified and evaluated by ssGSEA, the high-GBP5 cohort showed a higher immunomarker expression level, signifying the activation of infiltrating immune cells, notably including active CTLs, NK cells, and macrophages (**Figure 4E**).

The results of bioinformatic analyses above aligned with current research on GBP5. GBP5 has long been recognized as a proinflammatory mediator, frequently studied in the context of inflammatory diseases rather than tumor research 40-42. Its potential impact on OC development has remained unclear. Previous studies have consistently demonstrated a positive association between GBP5 expression and M1-type macrophage activation 40, 43, which potentially possessed anti-tumor capability by promoting immune response and inhibiting tumor growth. Our research further validates this conclusion, suggesting a potential avenue for translating GBP5's proinflammatory expertise into the field of tumor therapy.

**Figure 5:**
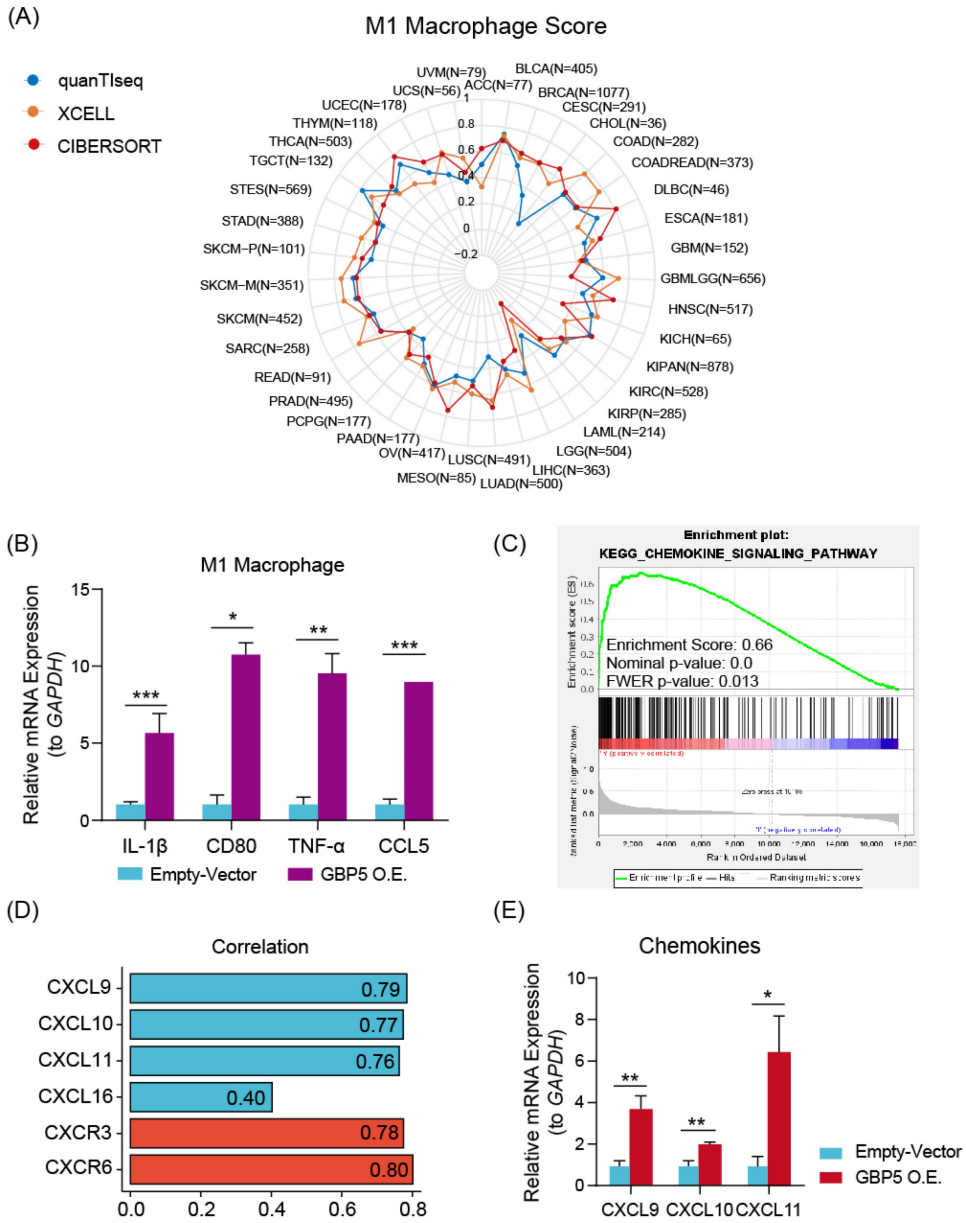
** (A)** The radar plot shows the distribution of Spearman correlation between M1-type macrophage infiltration and GBP5 expression in tumor types calculated by three distinct algorithms. The number of samples in specific cancer group was signed by “N”. The skin cutaneous melanoma (SKCM) was departed into primary (SKCM-P) and metastatic (SKCM-M) and showed in the radar plot. **(B)** qRT-PCR test showed the transcription level of M1 macrophage markers in cocultured Mø. “*” stands for p-Value < 0.05, “**” as p-Value < 0.01, “***” as p-Value < 0.001.** (C)** The enrichment plot of the chemokine signaling pathway exported by GSEA analysis. **(D)** Spearman correlation with the specific factors involved in the chemokine-chemokine receptors pathway gets graphed by the bar plot. CXCL family is colored blue, and the CXCR family is colored red.** (E)** qRT-PCR showed the transcription level of chemokines in GBP5-overexpressed HEY cells. “*” stands for p-Value < 0.05, “**” as p-Value < 0.01.

Various algorithms were utilized to evaluate the infiltrations of different subtypes of macrophages in 10180 tumor samples across diverse cancer types. Cancer types meeting following criteria were presented with corresponding Spearman correlation analysesl results: (I) Recorded in TCGA database; (II) By at least one kind of algorithm, the result showed statistical significance (P < 0.05). The full names of tumors were listed in **Table S1**. From the result of pan-cancer analysis, we found that GBP5 positively correlated with M1-type macrophage score in most types of cancer, except acute myeloid leukemia (LAML) when evaluated by CIBERSORT (**Figure 5A**), while the results of total macrophage score (**Figure S2A**) and M2-type macrophage scores (**Figure S2B**) were less correlated. When being assessed by CIBERSORT algorithm, the M2 macrophage infiltration even exhibited a negative correlation with GBP5 expression (**Figure S2B**). It could be concluded that the positive association between GBP5 expression and macrophage infiltration was universal pan-cancerly. Additionally, when being evaluated by ESTIMATE algorithm, we found that GBP5 expression closely associated with immune-score and ESTIMATE-score (**Figure S2C**).

We hypothesized that GBP5 affected M1 macrophage differentiation in a paracrine manner. Therefore, we established a coculture system comprising OC cells and macrophages. M0 macrophage cocultured with OC cells exhibited a decrese in the expressions of M2-type macrophage markers IL-10 and TGF-β1 (**Figure S2D**). M1 macrophages cocultured with OC cells exhibited a significant increase in M1 macrophage markers, including CD80, CCL5, IL-1β, and TNF-α (**Figure 5B**), indicating an elevation in M1 macrophage activation. GSEA analysis (**Figure 5C**) and Spearman correlation (**Figure 5D**) results stated that GBP5 potentially mediates immune cell recruitment through the modulation of chemokine pathways, specifically CXCL9/10/11-CXCR3 and CXCL16-CXCR6, in OC cells. qRT-PCR results also indicated that exogenous overexpression of GBP5 improved CXCL9/10/11 production in OC cells (**Figure 5E**).

Besides macrophage M1, GBP5 showed positive correlation with CD8^+^ T cell as well, referring from **Figure 4A**. The question remains whether this improved prognosis is attributed to increased T cell infiltration or M1 macrophages. To address this issue, we firstly overexpressed GBP5 in Jurkat T cells, and subsequently co-cultured untreated Jurkat cells with GBP5-overexpressing OC cells *in vitro*. Both groups of T cells were collected, and RNA was extracted for the detection of transcription levels of T cell activation markers, chemokine receptor, and tumor cytotoxicity effector molecules using rtqPCR. As a activation marker upregulated earliest following T cell activation, and the most commonly used biomarker for identifying antigen-responsive T cells in *in vitro* assays 44, CD69 expression was elevated in GBP5-overexpressed T cell, while IFNγ and GZMB (effector molecules mediating T cell cytotoxicity against tumor cells 45) expressions were downregulated significantly. Meanwhile, when co-cultured with GBP5-overexpressed HEY cells, the transcriptions of CCR5/7, which are crucial markers mediating T cell tumor infiltration 46, 47, were notably upregulated in T cell, simultaneously with a decrease in GZMB transcription (**Figure S2E, F**).

Collectively, elevated GBP5 expression in T cells potentially promotes T cell activation and infiltration, while simultaneously reducing the release of tumor-killing molecules, as evidenced by the downregulation of GZMB and IFNγ transcription in Jurkat cells. Given the dual role of GBP5 on effector T cells, it cannot be directly concluded that the improved prognosis of OC patients with higher GBP5 expression is solely due to increased T cell infiltration. To achieve a more definitive conclusion, we should prioritize the investigation of GBP5's capability to promote M1 macrophage polarization in subsequent analyses.

### High Expression of GBP5 Suppresses OC Progression

The earliest research to associate GBP5 with pyroptosis stated that, the exogenous expression of GBP5 in RAW 264.7 cells induced a heightened susceptibility to cell pyroptosis, which was accompanied by the activation of CASP1 48. Several mysteries still linger, such as whether the same conclusion can be reached in tumor cells, the specific form of pyroptosis GBP5 leads to, and the underlying mechanisms that remain to be uncovered.

**Figure 8:**
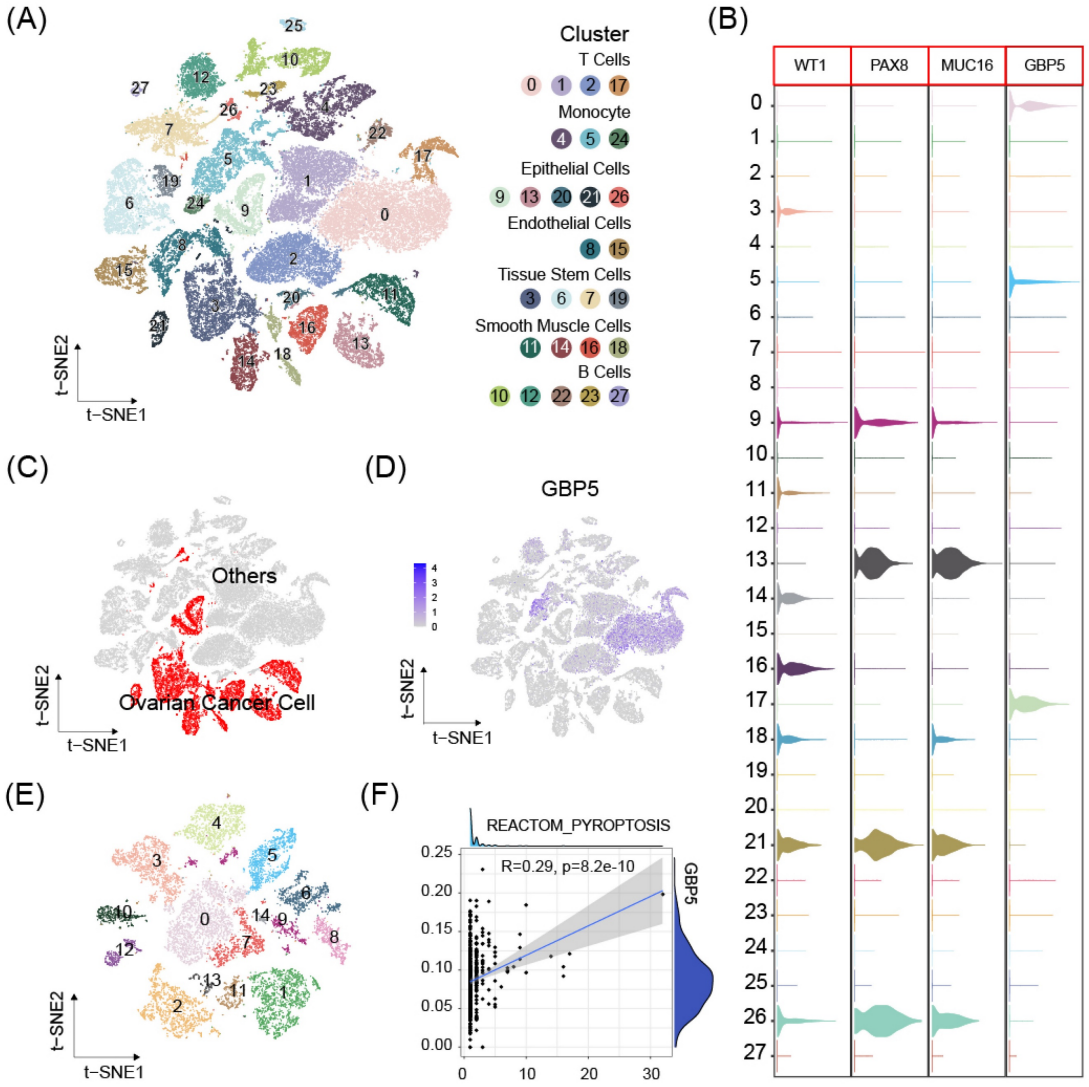
** (A)** The t-SNE plot of 51868 high-quality cells to visualize cell-type clusters based on the SingleR package. **(B)** Boxplot for the distribution of expression of the ovarian cancer marker WT1, PAX8, MUC16 and GBP5. **(C)** The t-SNE plot that showed the distribution of the cancer cell cluster (red, n = 13374) in the atlas. **(D)** The t-SNE plots show expression of GBP5. **(E)** Ovarian cancer cell populations were re-clustered into 15 subclusters.** (F)** The correlations between GBP5 expression (>0) and pyroptosis scores calculated by UCell algorithm in single cancer cells.

scRNA-seq data from 8 OC samples were downloaded from GEO database. Stringent criteria were applied to assess the quality of individual cells, to exclude low-quality or damaged cells that could introduce noise or bias into the analysis. The mitochondrial gene percent was controlled under 10% for each sample (**Figure S3A-D**). Dimensionality reduction using the t-distributed stochastic neighbor embedding (t-SNE) method and cell clustering were performed (**Figure S3E, 8A**). Cluster with high expressions of OC cell markers Wilms tumor 1 (WT1), Paired Box 8 (PAX8), Mucin 16 (MUC16) were defined as OC cell cluster 38 (**Figure 8B**). The expression of DUOX1, SERPINB1, TREM2 was either absent, or expressed in too few cells to be available for correlation analysis. GBP5 expression enriched in non-malignant cell clusters (**Figure 8C, D**), especially T cells, suggesting our aforementioned conclusion and high GBP5 expression may indicate limited cancer cell proliferation in tumor tissue. OC cell clusters extracted in previous single cell analysis were reclustered (**Figure 8E**), and further analysis by the Ucell algorithm revealed a positive correlation between the reactome pyroptosis score and GBP5 expression in single cells (only cells with GBP5 expression > 0 were included) (**Figure 8F**).

**Figure 9:**
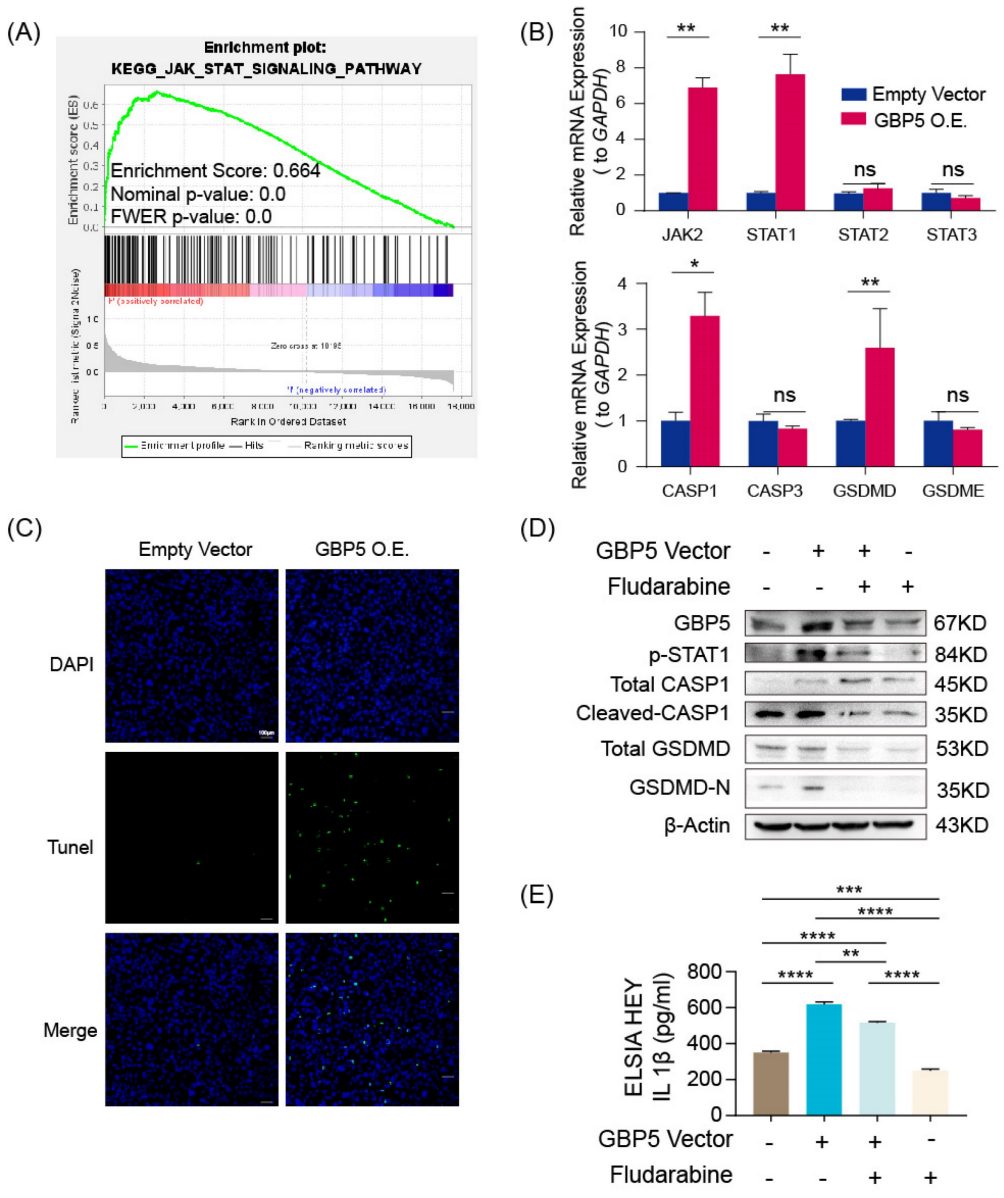
“GBP5 O.E.” stands for the cell cohort transfected by GBP5 overexpressed vector.** (A)** Gene set in the high-GBP5 group was enriched in the JAK-STAT pathway according to the result of GSEA.** (B)** Accompanied by GBP5 expression elevation, the transcription levels of JAK2, STAT1, CASP1, and GSDMD were upregulated, while STAT2, STAT3, CASP3, and GSDME had no significant change. (p-Value: ** p < 0.01, * p < 0.05, ns p > 0.05) **(C)** The exogenous overexpression of GBP5 improved the cell death rate tested by TUNEL staining; typical results were selectively shown at 10×. **(D)** Through western blotting, p-STAT1 expression was elevated in GBP5-overexpressed cohorts, while the cleavage and activation level of CASP1 and GSDMD increased while the total CASP1 slightly increased and total GSDMD remained steadily unchanged. The change brought by GBP5 exogenous overexpression can be blocked by STAT1 inhibitor fludarabine in rescue test. **(E)** The secretion of IL-1β was quantitatively detected by ELISA test in cell line HEY, as part of the rescue test. (p-Value: ** p < 0.01, *** p < 0.001, **** p < 0.0001)

According to the result of the GSEA analysis, the enrichment of genes in the JAK-STAT pathway in the high-GBP5 group offered a reasonable hypothesis (**Figure 9A**). As a crucial signaling pathway in PCD, it had been reported that increased JAK-STAT signaling (caused by JAK2^V617F^ mutation) fostered the formation of necrotic cores in atherosclerotic lesions, which can be reversed by the deletion of CASP1 or pyroptosis executioner GSDMD 49. It is reasonable to infer that JAK2/STAT is involved in the occurrence of cell pyroptosis.

In GBP5 highly-expressed HEY cells, we observed a significantly increased transcription level of JAK2 and STAT1, whereas the transcription levels of STAT2 and STAT3 remained steadily unchanged. The pyroptosis canonical pathway markers CASP1 and GSDMD transcription were also upregulated, while CASP3 (an apoptosis biomarker) and GSDME (an inflammasome-independent pyroptosis biomarker) hardly changed (**Figure 9B**). Demonstrated by TUNEL staining, GBP5 overexpression effectively increased cell death in OC cells (**Figure 9C**). To validate the proposed GBP5-JAK2-STAT1-CASP1-GSDMD axis, we divided HEY cells into 4 groups: GBP5 overexpressing-fludarabine group, GBP5 overexpressing-DMSO group, empty vectors-fludarabine group, empty vectors-DMSO group. The GBP5 overexpressing-DMSO group showed the highest expressions of GBP5, p-STAT1, and pyroptotic cell markers including cleaved-CASP1 and GSDMD-N with a slight increase in total CASP1 and unchanged expression of total GSDMD. In the rescue experiments, we inhibited STAT1 activation by using fludarabine, the GBP5 overexpressing-fludarabine and empty vector-fludarabine groups exhibited significant reductions in the protein expressions of p-STAT1 and activated canonical pyroptotic factors, compared with GBP5 overexpressing-DMSO and empty vector-DMSO groups, respectively. The same trend was observed in the IL-1β secretion, confirmed by ELISA (**Figure 9D, E**). Based on existing studies and experimental results, we concluded that, GBP5 facilitated pyroptotic cell death through JAK2-STAT1 pathway in OC cells.

### GBP5 Improved the Efficacy of Immunotherapy and Indicated a Better Post-Therapy Prognosis

As mentioned above, the significance of proinflammatory factors in tumor therapy lies in their potential to convert “cold tumors” into “hot tumors” in immunotherapy. It is crucial to explore whether GBP5 brings immunotherapy treatment benefits in OC.

**Figure 10:**
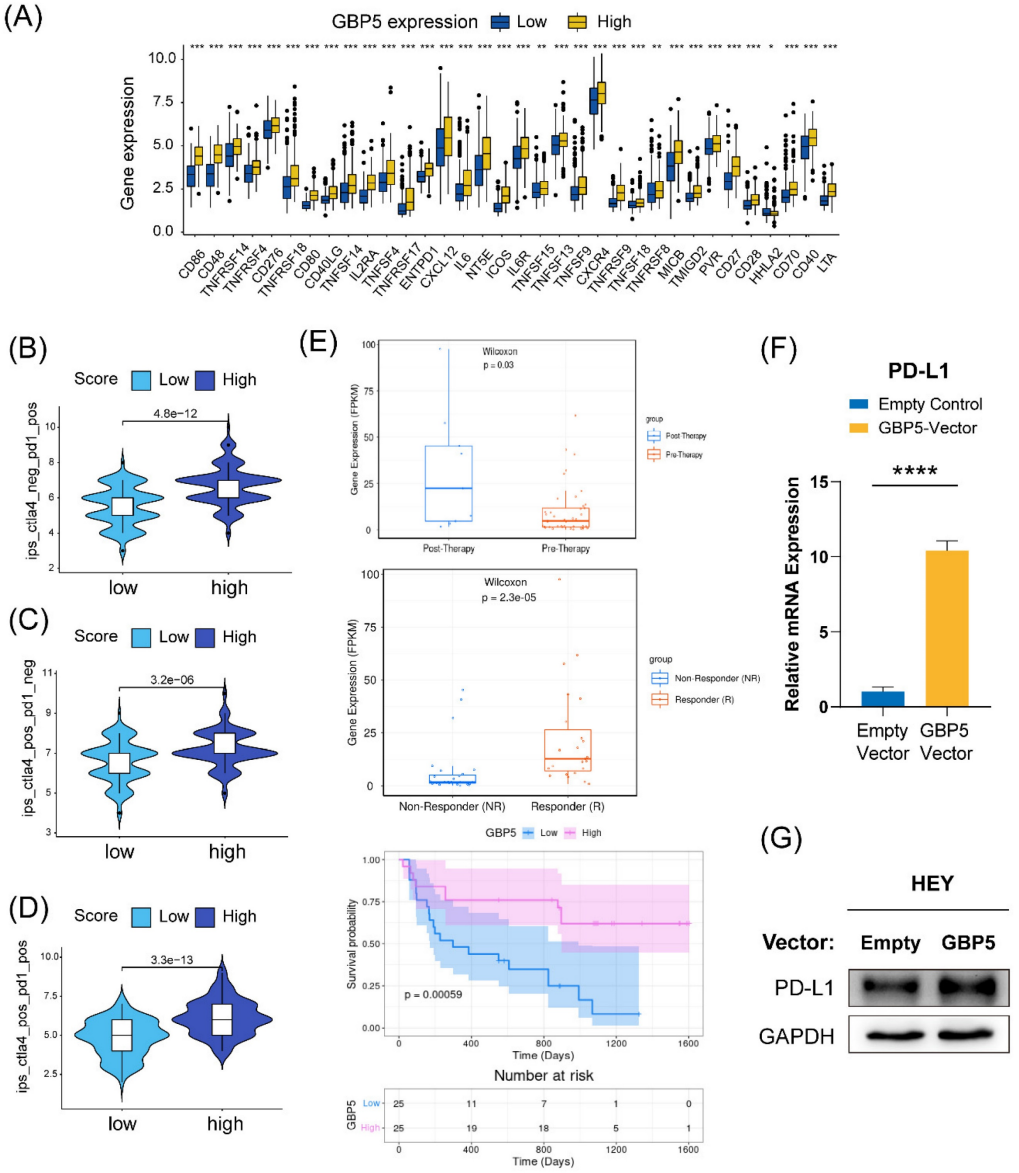
** (A)** ISCPs' expression concerning GBP5 was displayed. The GBP5 high expression group responded better under the operation of **(B)** immunotherapy with PD1 or** (C)** CTLA4 alone or a **(D)** combined application strategy.** (E)** The plots were downloaded from the TIGER database, showing information about GBP5 expression in an anti-PD1 cohort of melanoma patients.** (F)** The transcription and** (G)** expression levels of PD-L1 in HEY cells improved, “****” as p-Value < 0.0001.

We conducted a differential expression analysis of several immune-stimulating checkpoint proteins (ISCPs), revealing an increased expression in the high-GBP5 subgroup. Notably, the analyzed ISCPs included M1 macrophage markers CD86, CD80, members of the tumor necrosis factor (TNF) superfamily, and TNF receptor superfamily. These factors are crucial for the generation and function of TNF, which is produced by macrophages with tumor-killing effects. Additionally, chemokines and their corresponding receptors were also elevated in the high-GBP5 subgroup (**Figure 10A**). Referring from the distribution of IPS in OC patients, we predicted the responsivity of each OC patient to different strategies. Patient cohort with relatively high GBP5 expression tended to have better response to anti-PD1 and anti-cytolytic T lymphocyte-associated antigen (CTLA)4, no matter monotherapies (**Figure 10B, C**) or combined application (**Figure 10D**).

While there is currently no information on GBP5 in immunotherapy datasets of OC patients, data from research on melanoma patients receiving anti-PD-1 monotherapy is available. According to the Wilcoxon test, GBP5 expression levels were significantly higher in samples from anti-PD-1 responders (with a p-value < 0.0001) and post-anti-PD-1 immunotherapy patients (with a p-value = 0.03). Post-therapy, patients with high GBP5 expression exhibited better survival (**Figure 10E**), enhancing the reliability of IPS predictions. Through qRT-PCR and western blotting, we confirmed that PD-L1 transcription (**Figure 10F**) and expression (**Figure 10G**) were positively regulated by the overexpression of GBP5. The unprocessed original blots were showed in **Figure S4**. These findings suggest that GBP5 expression may provide a favorable environment for PD-L1 targeted strategies.

## Discussion

OC serves as a major cause of female death. The notable improved clinical prognosis for malignancies is due to the continuous advancements in medical technology, and the optimization of treatment protocols over recent decades 50. Compared to conventional chemotherapy, immunotherapy is a relatively novel therapy with rapid progression, experiencing remarkable evolution. It specifically targets human immune system. According to our research, OC patients can be divided into different molecular types, exhibiting varying TME status. In fact, immunotherapy effectiveness is also related to individual genetic molecular profile, that's why molecular typing plays crucial role in enhancing the efficacy of immunotherapy 51. Interaction between immune system and cancer microenvironment constructs TME 52. Changes occurring within the TME have been found to promote cancer immune evasion, ultimately leading to tumor progression and mortality. Immunotherapy interventions work to suppress or inhibit the immunosuppressive signals induced by the TME or tumor cells.

Cellular components of TME encompass various elements, including tumor cells, tumor-associated fibroblasts, endothelial cells, cytokines, growth factors, extracellular vesicles, Treg cells, CD8+ T cells, and other relevant immune cells 51, among these components, effector T cells, DC, NK, and M1-type TAMs are recognized as crucial anti-tumor immune cells. TAMs, being the predominant immune cell type within the TME, exhibit functional plasticity and can transition between pro-inflammatory M1 and anti-inflammatory M2 phenotypes 53. Besides, the type of cytokines produced within TME plays a critical role in determining tumor immunotherapy outcome, for their capability of influencing T cell infiltration and macrophage polarization. That's why we focused on pyroptosis, an inflammatory cell death form with pro-inflammatory molecules released. In this context, we introduced the potential role of the inflammatory factor GBP5 in the TME with significance. Not only due to its nature of pyroptotic cell death promoter in cancer cell, GBP5's capability of promoting M1 macrophage polarization potentially make the TME more immune-responsive, thus assisting the efficacy of immunotherapy. Through correlation analysis based on CIBERSORT scoring and the scRNA analysis, we also observed a correlation between enrichments of T cell and GBP5 high expression. However, it makes sense that the activation of M1 macrophage promotes the recruitment of effector T cells. Moreover, we conducted *in vitro* experiments to prove that, exogenous overexpression of GBP5 in T cells fails to augment their tumor-killing efficacy, while elevated GBP5 expression in OC cells does not directly enhance the secretion of GZMB and IFNγ from T cells.

Linking pyroptosis with M1 polarization of macrophages is not at all a forced association. Fatty acid-induced M1 polarization of macrophage leads to activation of CASP1-mediated classical pyrolysis pathway in pancreatic acinar cells 54, thus resulting in inflammation and pancreatic tissue damage. This process relied on cathepsin S-loaded exosome secreted by macrophage. Besides, in the context of cancer, a research on breast cancer 55 also indicated that, TAMs further promoted GSDME expression in dopamine receptor D2 (DRD2)-transfected BrCa cells during co-culture.

This suggests that the role of GBP5 in promoting M1 polarization of macrophages within the TME may further enhance its ability to induce OC cell pyroptosis, either through paracrine or other potential forms. However, in this study, we used to design a co-culture system where untreated OC cells were separately cultured with induced M1 macrophages and unpolarized M0 macrophages. In this process, pyroptotic cells were not observed in either group of OC cells. This may be due to the lack of specific components present in TME within *in vitro* environment, or it could be attributed to differences between commercially proliferated OC cell lines/THP1 cell line and primary human-derived cells. This represents a limitation of our study, and the underlying mechanisms require further exploration in future studies.

GBP5, the protagonist of this work, is generally believed to be associated with cell-autonomous immunity, involving in IFN-γ inducible inflammatory pathways. As a relatively newly discovered member of the GTPase superfamily, GBP5 still holds many mysteries that need to be uncovered 56, 57. However, there is limited research on GBP5 in the context of cancer. Despite being a member of dynamin, it surprisingly acts as a favorable indicator for the survival of OC patients, according to information obtained from TCGA and GEO databases. The underlying mechanism could be attributed to its proinflammatory and pyroptotic cell death-driving characteristics. There are instances where inducing pyroptosis has demonstrated a restraining impact on cancer progression 58, though it still remains intricate and dependent on specific circumstances. The first report to correlate GBP5 with pyroptosis discovered the capability of GBP5 to increase the sensitivity of RAW 264.7, a murine macrophage cell line, against Salmonella, by inducing pyroptotic cell death, and this process relies on GSDMD activation 48, 59. That's exactly why the current emphasis on GBP5 mostly centers around inflammatory diseases resulting from its heightened expression in macrophages, in some cases it could be identified as a M1-phenotype macrophage marker. 40 However, it's suggested that the capacity of Salmonella to induce pyroptosis extends beyond macrophages. Salmonella shows promise being applied in cancer immunotherapy. In a research about colorectal cancer immunotherapy, intravenous administration of Salmonella upregulated transcript levels of key molecules comprised within inflammasome signaling pathway in cancer cells. Moreover, when being intratumoral injected, Salmonella triggered inflammasome activation in melanoma cells, characterized by the surface exposure of GSDMD and calreticulin (CRT), along with the release of HMGB-1, indicative of immunogenic cell death, particularly pyroptosis 60. GBP5 may contribute to the development of enhanced Salmonella strains, potentially augmenting their antitumor efficacy, thus solving the primary challege of Salmonella-based cancer immunotherapy about the transient nature of its antitumor effect. Additionally, overexpression of GBP5 in cancer cells may enhance their susceptibility to Salmonella, thereby amplifying Salmonella's pro-pyroptotic effect. Moreover, M1 macrophages play a pivotal role in the antitumor activity of Salmonella. The efficacy of Salmonella-mediated melanoma cell killing diminishes significantly in the absence of inflammatory macrophage infiltration 60. From the perspective of optimizing the TIME, GBP5 also holds promise as an adjunct in Salmonella immunotherapy strategies. Further exploration of these avenues will be pursued in future investigations.

In our study, we also revealed a positive association between GBP5 and the JAK/STAT pathway, demonstrating that JAK2/STAT1 axis might participate in the regulation by GBP5 to OC cells pyroptosis. It has been reported that, phosphorylation and activation of JAK2/STAT1 are inextricably linked to the initiation of pyroptosis. JAK-STAT activating mutation of JAK2 (JAK2^V617F^) promotes the formation of necrotic cores in atherosclerotic plaques, and this effect can be reversed by the deletion of CASP1 or the pyroptosis executor GSDMD 49. Additionally, STAT1 has been considered the most promising transcription factor for GSDMD inferred from online databases. It has been found that STAT1 directly binds to the promoter region of GSDMD in a phosphorylated form by tyrosine 701 (Tyr 701) residue and drives the transcription of GSDMD, thereby promoting the pyroptosis of renal tubular epithelial cells during acute kidney injury 61. The phosphorylation of JAK2/STAT1 also aggravates the activation of NOD-like receptor thermal protein domain associated protein (NLRP) 3 inflammasome resulting in microglial pyroptosis, and such pro-pyroptotic function can be abolished by JAK2 inhibitor 62. In summary, our research results collectively indicate that GBP5 is an effective potential target for inducing pyroptosis in OC cells, and JAK2/STAT1 plays a crucial role in this process.

However, whether GBP5 is involved in the modulation of inflammatory microenvironment in OC remains undisclosed. Through coculture experiments, we found that OC cells with high expression of GBP5 secreted high levels of chemokines such as CXCL9/10/11, promoting the polarization of the co-cultured polarized M1-type macrophages. The chemokine-chemokine receptor signaling pathway plays a vital role in the recruitment of immune cells. Previous studies have indicated that the high secretions of CXCL9/10/11 by tumor cells imposes a specific recruiting effect on CXCR3^+^ CTL, NK cells, and macrophages 63. Moreover, these chemokines positively mediated the polarization of proinflammatory classical macrophages. JAK-STAT axis plays a role in the production of chemokines, similar to the observed phenomenon in macrophages and human mesangial cells 64. Research has shown that JAK2/STAT1 can promote the transcription of CXCL10, and selective JAK2 inhibitors can block this process 65. Comprehension of these reports provided an acceptable explanation for the phenomenon that OC cells with high expression of GBP5 can promote the polarization of cocultured M1 macrophages.

Previous research has shown a correlation between elevated PDL1 expression and high GBP5 levels, particularly in human glioma 57. This association invests PDL1 with a positive role in the prognosis of cancer patients receiving anti-PD-1/PD-L1 therapy. Additionally, there are records indicating an increase in PDL1 expression correlated with the activation of JAK2-STAT1 after IFN-γ induction. Foretinib treatment has been shown to upregulate the JAK2-STAT1 axis, resulting in increased PD-L1 expression and improved responsivity to ICIs when combined with anti-PD-1 antibody 66. It offers another possible way for GBP5 to involve in improving immunotherapy efficacy.

Overall, our study reveals that, GBP5 predicts an immunoreactive TME in OC patients, as well as promotes OC cell pyroptosis and the polarization of M1 macrophage potentially through the activation of JAK2-STAT1 and chemokines secretion. Involved factors above potentially reinforce each other, creating a positive feedback loop with stronger immune responsivity, providing new insights for improving OC immunotherapy efficacy.

## Conclusion

In this research, we discovered a benign biomarker GBP5 in OC, which was commonly considered as an inflammatory modulator and hardly correlated with cancer research. We further researched the underlying mechanism of GBP5's pyroptosis-inducing effect via JAK2-STAT1 pathway in OC cells, and its function on recruiting immune cells, enhancing the polarization of M1 macrophage by promoting the production of chemokines. The proinflammatory characteristic of GBP5 shows vital potential for being applied in immunotherapy.

## Supplementary Material

Supplementary figures and tables.

## Figures and Tables

**Figure 6:**
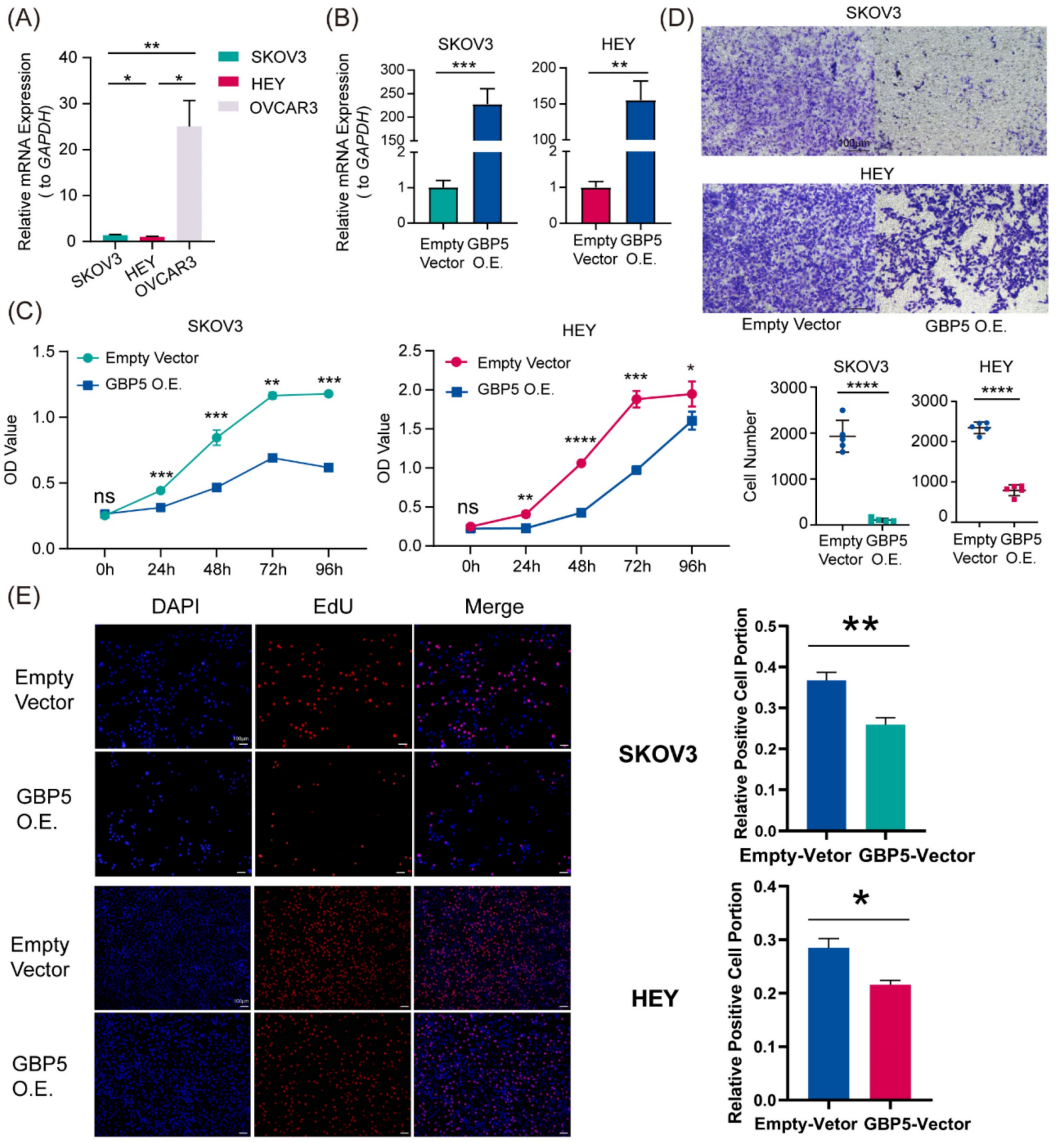
“GBP5 O.E.” stands for the cell cohort transfected by GBP5 overexpressed vector. **(A, B)** qRT-PCR showed (**A**) the original transcription level of GBP5 in three OC cell lines and (**B**) the efficacy of transfection. “*” stands for p-Value < 0.05, “**” as p-Value < 0.01, “***” as p-Value < 0.001. **(C)** The OC cell viability got measured by OD value in CCK8 assay (p-Value: *** p < 0.001 ** p < 0.01, * p < 0.05, ns p > 0.05).** (D)** We selectively show typical results of the Transwell assay conducted on SKOV3 and HEY cell lines; in each group, we observed the chamber bottom under 10× stereomicroscope and randomly choose five visual fields and perform cell counting through Image J software. The difference in the invaded cell number between the two groups was taken into statistics (p-Value: **** p < 0.0001). **(E)** EdU assay was used to detect the proliferation rate in two hours of each group of SKOV3 and HEY cells. Images were taken under 10× fluorescence microscope. Scale bar, 100 μm. Positive cells exhibited red fluorescence staining, while the nuclei were fluorescently stained in blue. “*” stands for p-Value < 0.05, “**” as p-Value < 0.01.

**Figure 7:**
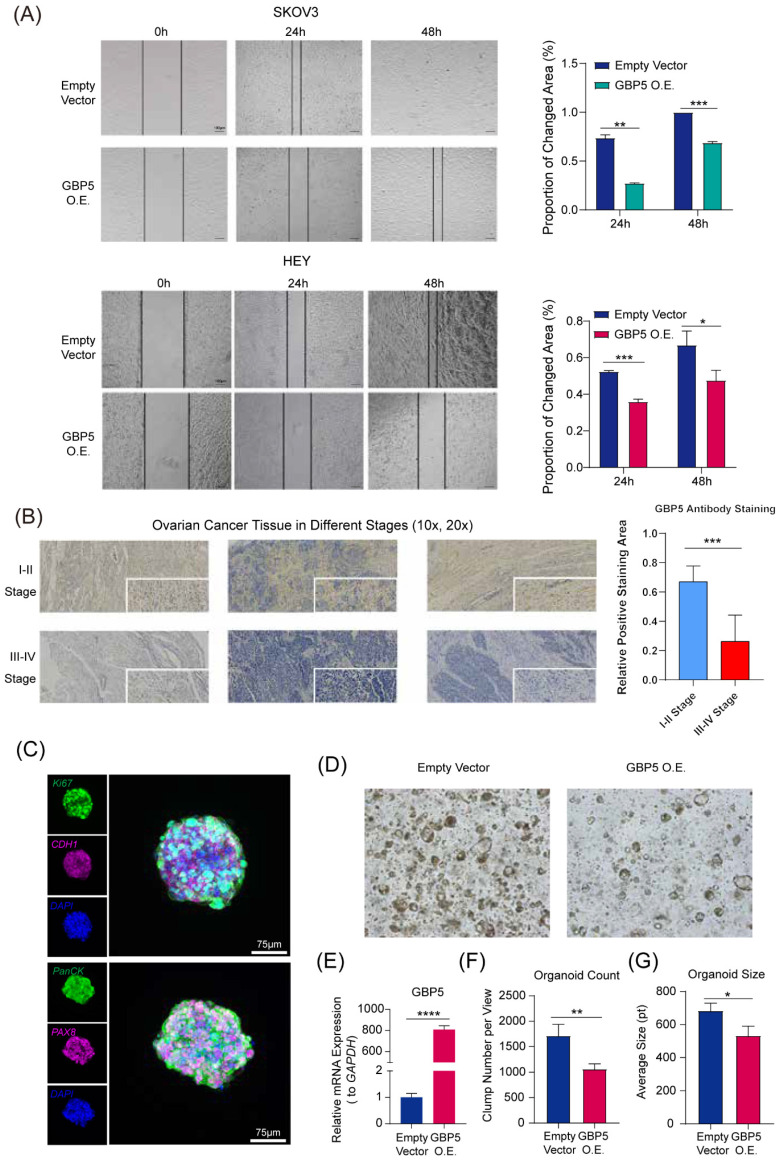
“GBP5 O.E.” stands for the cell cohort transfected by GBP5 overexpressed vector.** (A)** The microscopy views were graphed at 12 hours, 24 hours, and 48 hours after making the scratches. The blank areas were calculated through Image J, and the proportion of changed area got exhibited as the indicator of OC cell migration. (p-Value: *** p < 0.001, ** p < 0.01, * p < 0.05) **(B).** The immunohistochemical staining results of GBP5 in HGSOC tissue. GBP5 positive expression in the OC tissues was mainly located around the cell cytoplasm and higher in the samples of FIGO I-II stages. The outcome of differential analysis based on positive staining area proportions were with statistical significance. (p-Value: *** p < 0.001)** (C)** Multiplex immunofluorescence profiling of OC organoids. DAPI (blue) staining highlights the cellular architecture of the organoids. PAX8 (magenta) and PanCK (green) highlights the presence of specific ovarian cancer cell populations. Ki67 and CDH1 were respectively stained in green and magenta. **(D)** The Matrigel droplets containing OC organoids got observed under a 5× microscope, and typical results were selectively shown.** (E)** qRT-PCR showed the transfection efficiency of GBP5-vector to OC organoids, **(F)** the average organoid count in each view and **(G)** the size of clumps was taken into statistics. “*” stands for p-Value < 0.05, “**” as p-Value < 0.01, “***” as p-Value < 0.001, “****” as p-Value < 0.0001.

## Abbreviations

CASP: caspase
CCL: C-C motif chemokine ligand
CCR: C-C motif receptor
CXCL: C-X-C motif chemokine ligand
DC: dendritic cell
DEG: differentially expressed genes
EOC: epithelial ovarian cancer
GAPDH: glyceraldehyde-3-phosphate dehydrogenase
GBP: guanylate-binding protein
GSDMD-N: N-terminal in gasdermin D
GSEA: gene set enrichment analysis
GZMB: Granzyme B
ICI: immune checkpoint inhibitor
IFN: interferon
IHC: immunohistochemistry
IPS: immunophenoscore
ISCP: immune-stimulating check point
JAK: janus kinase
NK: natural killer
NLRP: NOD-like receptor thermal protein domain associated protein
OC: ovarian cancer
OS: overall survival
PAC: proportion of ambiguous clustering
PCD: programmed cell death
PDL: programmed cell death 1 ligand
PDOCO: patient-derived ovarian cancer organoid
STAT: signal transducer and activator of transcription
TAM: tumor-associated macrophage
TCR: T cell receptor
TIME: tumor immune microenvironment
