# Supplementary material for: Immunoreactive Microenvironment Modulator GBP5 Suppresses Ovarian Cancer Progression by Inducing Canonical Pyroptosis
Source: J Cancer. 2024 May 5;15(11):3510–30. doi: 10.7150/jca.94616 (PMC11134437; doi:10.7150/jca.94616)
Supplement: Supplementary file 1 — Supplementary figures and tables. [file jcav15p3510s1.pdf]

Supplementary Material

Supplementary Figure

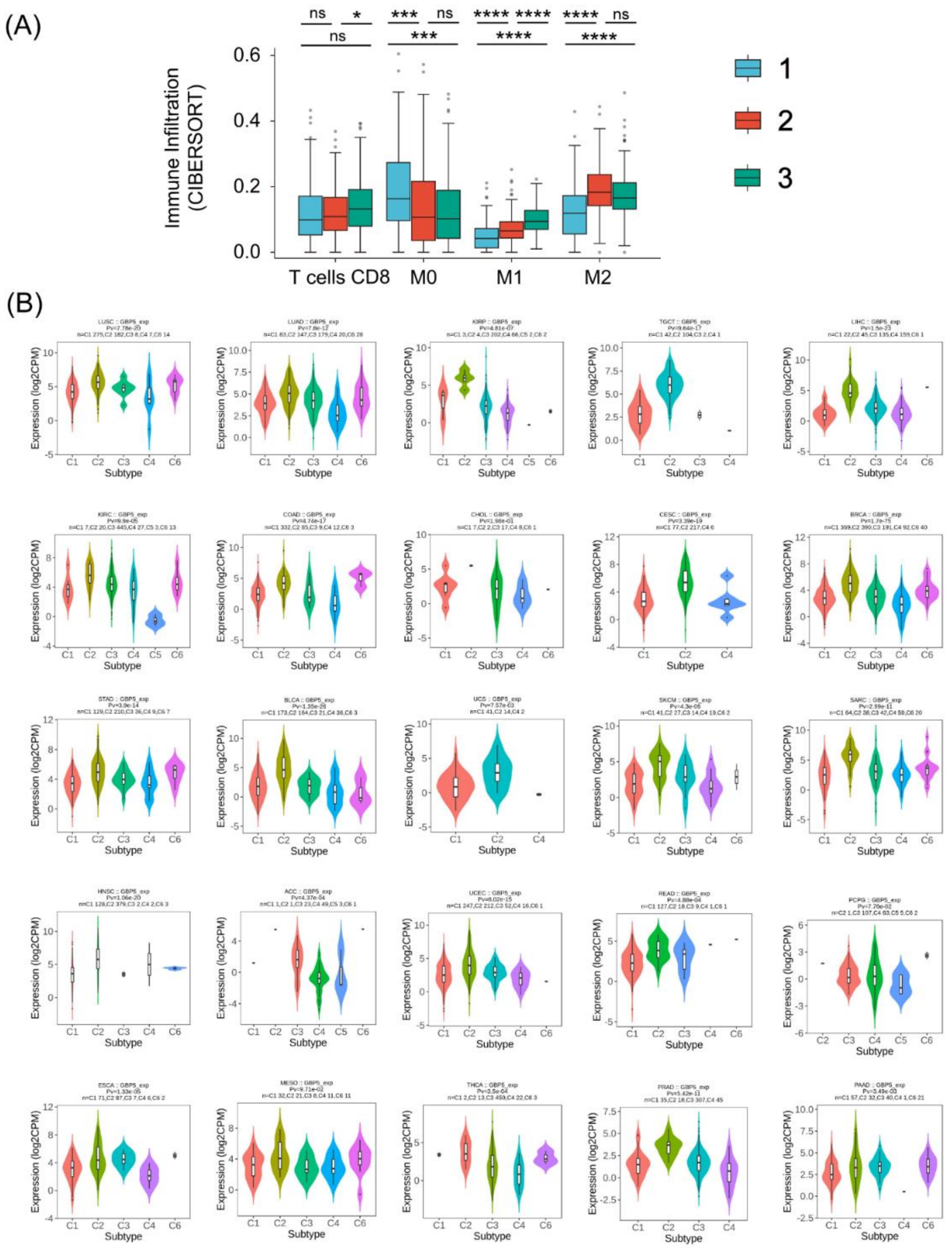

Figure S1. (A) Infiltration scores of various immune cells in tumor tissues for ovarian cancer patients grouped based on non-negative matrix factorization (NMF) (Group 1, 2, 3), using the CIBERSORT algorithm. (p-Value: ns $p > 0.05$, * $p < 0.05$, *** $p < 0.001$, **** $p < 0.0001$) **(B)** GBP5 expression level of various immune subtypes in pan-cancer.

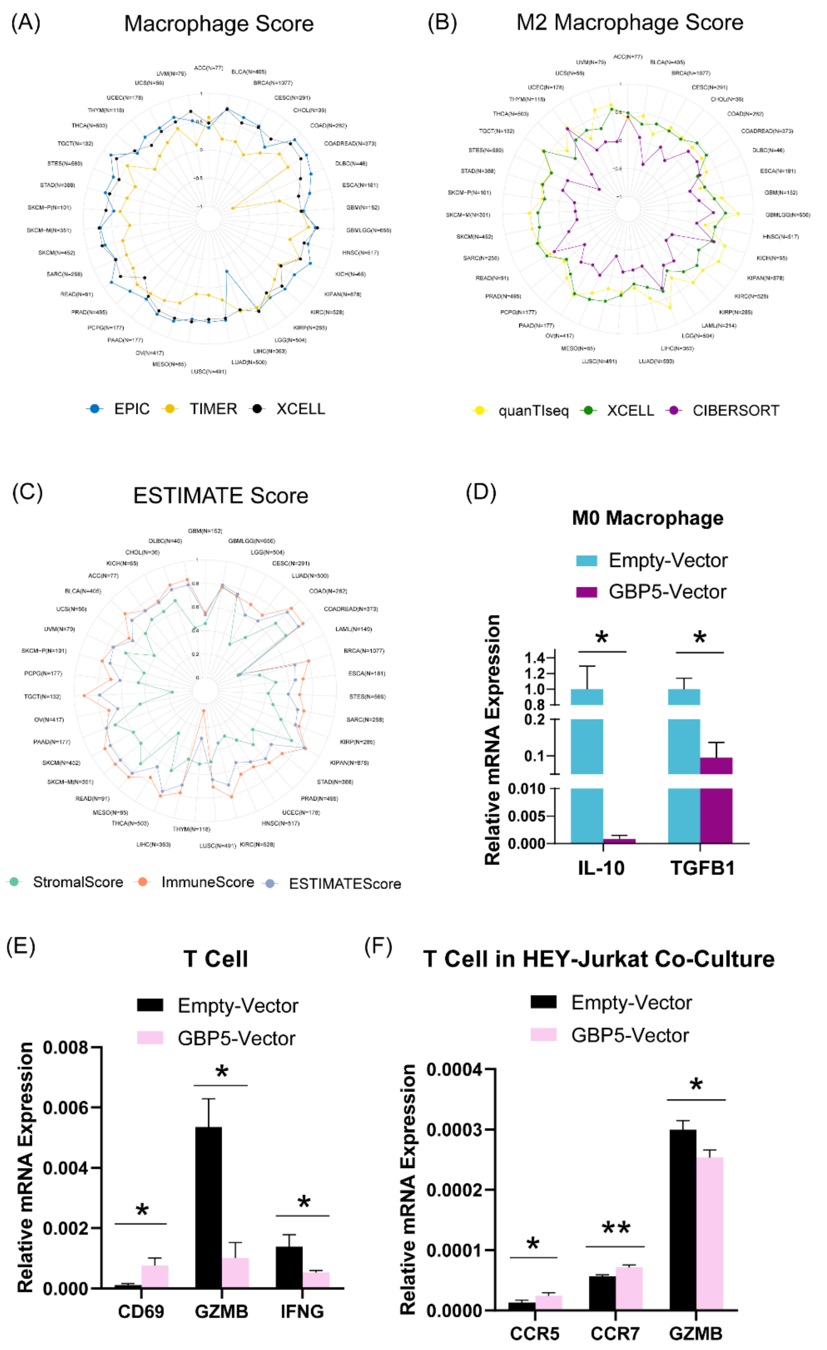

Figure S2. The radar plots respectively show the distribution of Spearman association of **(A)** macrophage scores, **(B)** M2 macrophage scores, and **(C)** ESTIMATE scores with GBP5 expression in various tumor types calculated by three distinct algorithms. The number of samples in specific cancer group was signed by “N”. The skin cutaneous melanoma (SKCM) was departed into primary (SKCM-P) and metastatic (SKCM-M) and showed in the radar plot. **(D)** qRT-PCR test showed the transcription level of M0 macrophage markers in cocultured Mø. **(E)** qRT-PCR test showed the transcription level of T cell activation marker (CD69) and tumor killing molecules (GZMB, IFNγ) in T cells, as well as **(F)** chemokine receptors (CCR5, CCR7) and GZMB in T cells cocultured with OC cells. "*" stands for p-Value < 0.05, while "**" stands for p-Value < 0.01.

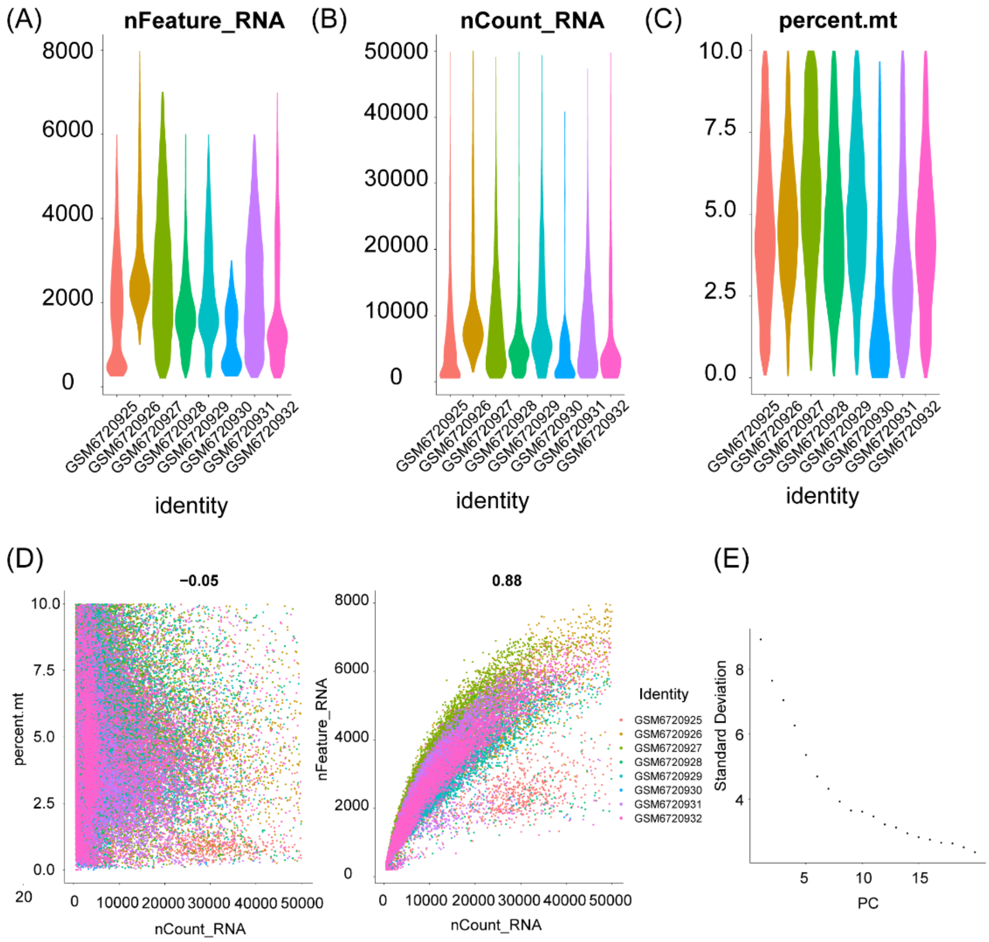

Figure S3. The quality control step of the scRNA analysis. **(A)** "nFeature_RNA" represents the number of genes detected in each cell, cells with fewer than 200 measured genes were excluded. To delete cells with extraordinary gene number in each sample, we excluded cells with nFeature > 6000 in GSM6720925 (9804 cells), GSM6720928 (9223 cells), GSM6720929 (5277 cells), and GSM6720931 (6229 cells), > 7000 in GSM6720927 (7277 cells) and GSM6720932 (8523 cells), > 8000 or <1000 in GSM6720926 (5296 cells), > 3000 in GSM6720930 (239 cells). **(B)** "nCount" represents the sum of expression levels for all genes detected in each cell, cells with a total expression amount exceeding 50,000 are removed from the dataset. **(C)** "percent.mt" represents the proportion of mitochondrial genes detected, the mt-percent of each cell was kept lower than 10%, for the sample originated from human ovaries. **(D)** The scatter plots show the association of mitochondrial gene percentage (left) and feature number (right) with the total expression amount respectively, the title numbers stand for the R values. **(E)** The Jackstraw plot.

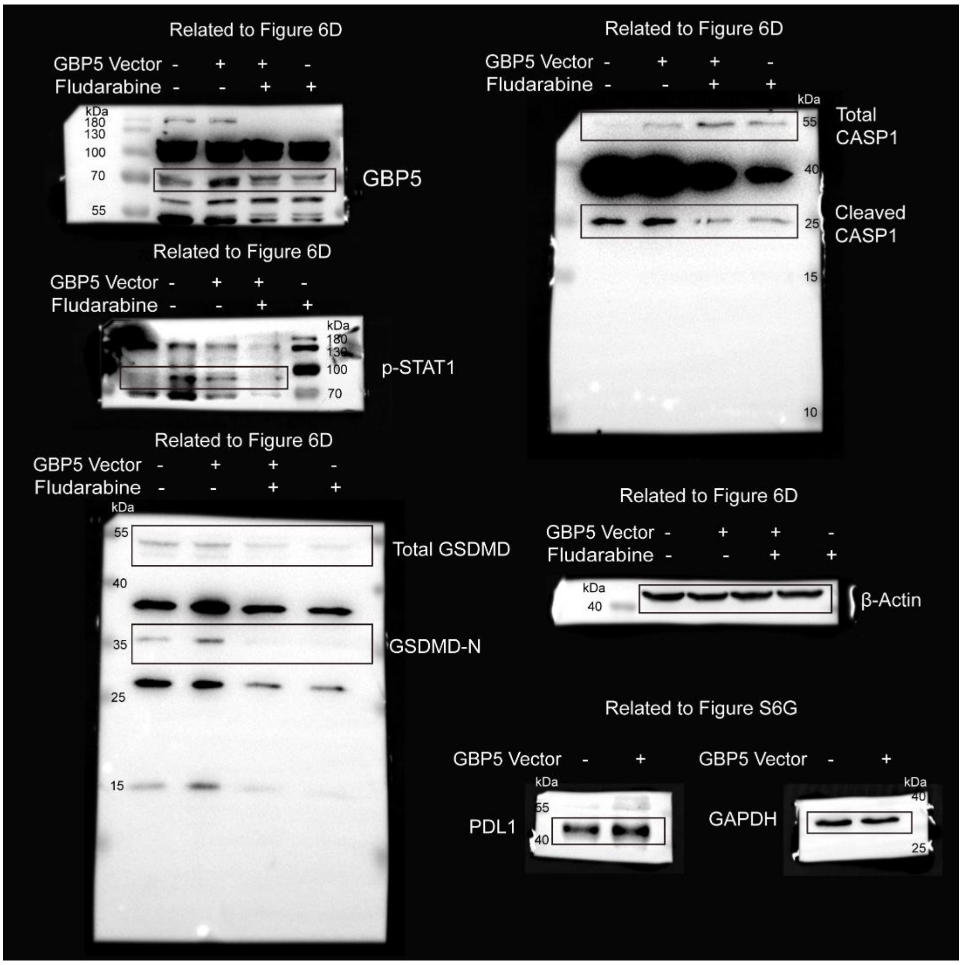

Figure S4. The unprocessed original blot of the western blotting. Parts of the image cited in the manuscript has been labeled.

Supplementary Tables

Table S1. Tumor types included in the pan-cancer analysis.

Cohorts	Names of cancer types
ACC	Adrenocortical carcinoma
BLCA	Bladder Urothelial Carcinoma
BRCA	Breast invasive carcinoma
CESC	Cervical squamous cell carcinoma and endocervical adenocarcinoma
CHOL	Cholangiocarcinoma
COAD	Colon adenocarcinoma
COADREAD	Colon adenocarcinoma/Rectum adenocarcinoma Esophageal carcinoma
DLBC	Lymphoid Neoplasm Diffuse Large B-cell Lymphoma
ESCA	Esophageal carcinoma
FPPP	FFPE Pilot Phase II
GBM	Glioblastoma multiforme
GBMLGG	Glioma
HNSC	Head and Neck squamous cell carcinoma
KICH	Kidney Chromophobe
KIPAN	Pan-kidney cohort (KICH+KIRC+KIRP)
KIRC	Kidney renal clear cell carcinoma
KIRP	Kidney renal papillary cell carcinoma
LAML	Acute Myeloid Leukemia
LGG	Brain Lower Grade Glioma
LIHC	Liver hepatocellular carcinoma
LUAD	Lung adenocarcinoma
LUSC	Lung squamous cell carcinoma
MESO	Mesothelioma
OV	Ovarian serous cystadenocarcinoma
PAAD	Pancreatic adenocarcinoma
PCPG	Pheochromocytoma and Paraganglioma
PRAD	Prostate adenocarcinoma
READ	Rectum adenocarcinoma
SARC	Sarcoma
STAD	Stomach adenocarcinoma
SKCM	Skin Cutaneous Melanoma
STES	Stomach and Esophageal carcinoma
TGCT	Testicular Germ Cell Tumors
THCA	Thyroid carcinoma
THYM	Thymoma
UCEC	Uterine Corpus Endometrial Carcinoma
UCS	Uterine Carcinosarcoma
UVM	Uveal Melanoma

Table S2. Primer nucleotide sequence of this study.

Gene	Primer nucleotide sequence
GAPDH	Forward: 5’-CTGGGCTACACTGAGCACC-3’
	Reverse: 5’-AAGTGGTCGTTGAGGGCAATG-3’
GBP5	Forward: 5’-TGCTATCGACCTACTGCACAA-3’
	Reverse: 5’-GCAGGATCTTCAACCCTGTCA-3’
JAK2	Forward: 5’-TCTGGGGAGTATGTTGCAGAA-3’
	Reverse: 5’-AGACATGGTTGGGTGGATACC-3’
STAT1	Forward: 5’-CAGCTTGACTCAAAATTCCTGGA-3’
	Reverse: 5’-TGAAGATTACGCTTGCTTTTCCT-3’
STAT2	Forward: 5’-CCAGCTTTACTCGCACAGC-3’
	Reverse: 5’-AGCCTTGGAATCATCACTCCC-3’
STAT3	Forward: 5’-CAGCAGCTTGACACACGGTA-3’
	Reverse: 5’-AAACACCAAAGTGGCATGTGA-3’
CASP1	Forward: 5’-TTTCCGCAAGGTTCGATTTTCA-3’
	Reverse: 5’-GGCATCTGCGCTCTACCATC-3’
CASP3	Forward: 5’-CATGGAAGCGAATCAATGGACT-3’
	Reverse: 5’-CTGTACCAGACCGAGATGTCA-3’
GSDMD	Forward: 5’-GTGTGTCAACCTGTCTATCAAGG-3’
	Reverse: 5’-CATGGCATCGTAGAAGTGGAAG-3’
GSDME	Forward: 5’-TGCCTACGGTGTCATTGAGTT-3’
	Reverse: 5’-TCTGGCATGTCTATGAATGCAAA-3’
IL10	Forward: 5’-TCAAGGCGCATGTGAACTCC-3’
	Reverse: 5’-GATGTCAAACTCACTCATGGCT-3’
TGFB1	Forward: 5’-CTAATGGTGGAAACCCACAACG-3’
	Reverse: 5’-TATCGCCAGGAATTGTTGCTG-3’
IL1B	Forward: 5’-CCACAGACCTTCCAGGAGAATG-3’
	Reverse: 5’-GTGCAGTTCAGTGATCGTACAGG-3’
CD80	Forward: 5’-CTCTTGGTGCTGGCTGGTCTTT-3’
	Reverse: 5’-GCCAGTAGATGCGAGTTTGTGC-3’
TNF-α	Forward: 5’-CCCGACTATCTCGACTTTGC-3’
	Reverse: 5’-AAGGTTGGATGTTCGTCCTC-3’
CCL5	Forward: 5’-ATATGGCTCGGACACCACTC-3’
	Reverse: 5’-TTCTTCGAGTGACAAACACG-3’
CXCL9	Forward: 5’-TCTGATTGGAGTTCAAGGAGC-3’
	Reverse: 5’-CATGTTTGGTCTCCATTCTTCA-3’
CXCL10	Forward: 5’-GACTCTGAGTGGAACTCAAGGAAT-3’
	Reverse: 5’-GTGGCAATGATCTCAACACG-3’
CXCL11	Forward: 5’-TGTGCTACAGTTGTTCAAGGCTTCC-3’
	Reverse: 5’-CTTGCTTGCTTCGATTTGGGATTTAGG-3’
CD69	Forward: 5’-ATTGTCCAGGCCAATACACATT-3’
	Reverse: 5’-CCTCTCTACCTGCGTATCGTTTT-3’
GZMB	Forward: 5’-CCCTGGGAAAACACTCACACA-3’
	Reverse: 5’-GCACAACTCAATGGTACTGTCG-3’

Gene	Primer nucleotide sequence
IFNG	Forward: 5’-TCGGTAACTGACTTGAATGTCCA-3’
	Reverse: 5’-TCGCTTCCCTGTTTTAGCTGC-3’
CCR5	Forward: 5’-TTCTGGGCTCCCTACAACATT-3’
	Reverse: 5’-TTGGTCCAACCTGTTAGAGCTA-3’
CCR7	Forward: 5’-TGAGGTCACGGACGATTACAT-3’
	Reverse: 5’-GTAGGCCCACGAAACAAATGAT-3’

Table S3. The composition of ovarian cancer organoid culture medium.

Reagent	Concentration	Ultimate Density	Per 10ml
Advanced D/F12			
HEPES	100×	10mM	100μl
GlutaMAX	100×	1×	100μl
P/S	100×	1×	100μl
Primocin		50mg/ml	20μl
B27	50×	1×	200μl
EGF	100μg/ml	5ng/ml	0.5μl
FGF10	20μg/ml	10ng/ml	5μl
A8301	10mM	500nM	0.5μl
Noggin	100μg/ml	100ng/ml	10μl
R-spondin1	500μg/ml	400ng/ml	8μl
Wnt3a	40μg/ml	20ng/ml	5μl
Y-27632	10mM	5μM	5μl
Heregulinβ-1	50μg/ml	37.5ng/ml	7.5μl
Nicotinamide	1M	10mM	100μl
N-Acetylcysteine	0.5M	1.25mM	25μl
17-β Estradiol	100μM	100nM	10μl
Hydrocortisone	100μg/ml	500ng/ml	5μl
Forskolin	25mM	10μM	4μl
